# The second decade of DTI in TBI Part 2: a systematic review of moderate and severe TBI

**DOI:** 10.3389/fneur.2026.1734550

**Published:** 2026-02-06

**Authors:** Molly F. Charney, Simone Glajchen, Shawn Brain, Fahmida Rashid, Sabrina Kentis, Melvin Alexander, Arvind Dev, Jenasis Ortega, Chihiro Okada, Brian Morris, Timothy Darby, Taskin Forkan, Anthony D. Yao, Yuchen Dong, Cindy Zhou, Emily Hunt, Jane Wee, Caroline Delbourgo Patton, Michael L. Lipton

**Affiliations:** 1 Department of Neurology, Columbia University Irving Medical Center, New York, NY, United States; 2 Department of Radiology, Columbia University Irving Medical Center, New York, NY, United States; 3 Touro College of Osteopathic Medicine, New York, NY, United States; 4 Department of Radiology, University of California Los Angeles, Los Angeles, CA, United States; 5 Albert Einstein College of Medicine, Bronx, NY, United States; 6 Department of Neurology, New York University Langone Hospital, New York, NY, United States; 7 Department of Radiology, Lennox Hill Hospital, New York, NY, United States; 8 Department of Radiology, Montefiore Medical Center, New York, NY, United States; 9 Albert Einstein College of Medicine, D. Samuel Gottesman Library, Bronx, NY, United States; 10 Department of Biomedical Engineering, Columbia University, New York, NY, United States

**Keywords:** cognition, DTI, neuroimaging, review, TBI

## Abstract

**Background:**

Traumatic Brain Injury (TBI) is a pervasive and important public health concern. TBI can range from mild, resulting in headaches and other neurologic symptoms, to severe resulting in coma and death. Diffusion tensor imaging (DTI) offers the ability to assess tissue microstructure at a level inaccessible to classical neuroimaging methods, such as CT and structural MRI. This systematic review aims to explore studies using DTI in moderate–severe TBI (msTBI) during the 2012–2022 decade, which is the second decade of reported use. The use of DTI in mild TBI during this time period is discussed in our companion systematic review.

**Methods:**

A systematic literature review was conducted by a medical librarian in accordance with the Preferred Reporting Items for Systematic Reviews and Meta-Analyses (PRISMA) guidelines. We searched the electronic databases PubMed/MEDLINE, Embase, Cochrane Library, and Web of Science from 2012 through September 28, 2022.

**Results:**

One hundred twenty-nine studies of moderate to severe TBI were included, which accounts for 9,609 patients. There were more longitudinal studies in 2012–2022 compared to the prior decade (25.6% vs. 13%). Fractional anisotropy (FA) and mean diffusivity (MD) were the most commonly used DTI measures. Regardless of acquisition techniques and analysis methods, the majority of studies that compared FA between those with msTBI and controls, found lower FA in msTBI patients. Lower FA was associated with worse cognitive outcomes and greater severity of TBI.

**Conclusion:**

Since its first decade (2002–2012) of reported use, DTI applications to msTBI have continued to expand in both quantity and scope, including notable increases in longitudinal studies, those employing whole brain analyses, and those addressing clinical and cognitive outcomes. The most salient finding across studies remains similar to 2002–2012, that despite heterogeneity of clinical and technical features of the individual studies, lower FA is consistently identified in msTBI patients compared to controls.

**Systematic Review Registration:**

https://www.crd.york.ac.uk/prospero, identifier CRD42022361318.

## 1 Introduction

Over 5.5 million people sustain moderate or severe traumatic brain injury (msTBI) each year worldwide (1). msTBI, commonly due to motor vehicle collision (MVCs), falls, and assaults (2), is a leading cause of mortality and morbidity across the lifespan. Prognosis following injury varies considerably, with msTBI frequently associated with prolonged hospitalization, extensive rehabilitation requirements, and diminished quality of life (3). While there has been significant advancement in the care and management of patients with msTBI, prognostic indicators and therapeutic interventions for msTBI remain extremely limited.

TBI classification is based on mechanism (closed vs. penetrating) and acute clinical severity assessment (e.g., Glasgow Coma Scale). Neuroimaging offers an objective assessment of injury in patients diagnosed with msTBI. Gross injury to the brain, detectable on CT and structural MRI is a common feature of msTBI and is a key target of acute management (4). Gross imaging findings, however, do not reliably predict outcome or recovery (4). Diffusion tensor imaging (DTI) offers the ability to assess tissue microstructure at a level inaccessible to CT and structural MRI (5). Animal and human studies have shown it is well-suited to the characterization of traumatic axonal injury (5). Since its first published application to TBI in 2002, DTI has been used extensively to characterize white matter effects of TBI (6). A comprehensive systematic review reported on studies applying DTI to TBI from 2002 to 2012, its first decade of reported use (6). The present systematic review encompasses published studies of DTI applied to msTBI during 2012–2022. Due to the large number of DTI studies on TBI published since 2012, we report separately on studies of mild TBI (mTBI) in a companion paper. We present here a comprehensive review of 129 studies, describe how this landscape has changed from the previous decade; and compare msTBI findings to those reported in mTBI.

## 2 Materials and methods

### 2.1 Protocol and registration

The protocol for this systematic review was registered in Prospero (CRD42022361318) and is available online www.crd.york.ac.uk/prospero/display_record.php?RecordID=361318.

### 2.2 Literature review

A systematic literature review was conducted by a medical librarian in accordance with the Preferred Reporting Items for Systematic Reviews and Meta-Analyses (PRISMA) guidelines (7). We searched the electronic databases PubMed/MEDLINE, Embase, Cochrane Library, and Web of Science on September 28, 2022. A combination of controlled vocabulary and text words was used. Terms included: “diffusion tensor imaging,” “DTI,” “traumatic brain injur*,” “TBI,” and “concussion.” The searches were conducted without any geographical restrictions and were limited to English-language articles only. Only articles published between 2012 and the date of our search, September 28, 2022, were included since the purpose of this review was to update a previously published review focusing on 2002–2012. Complete search strategy is included in the Supplemental materials.

### 2.3 Study selection

All references were imported into Endnote 20 reference management software (Clarivate, Philadelphia, PA) and de-duplication was carried out. They were then uploaded to Covidence (Veritas Health Innovation, Melbourne, Australia), an online literature review management tool. Further de-duplication was performed, followed by screening of the articles against the eligibility criteria, first based on the title and abstract and then based on the full text. Each article was independently assessed by two reviewers who were blinded to each other’s decisions. Conflicts were resolved by the lead reviewers (MC and FR). Details of the article screening and key decisions were preserved in Covidence. Studies were included in the systematic review if they met the following criteria: (1) peer-reviewed original research; (2) written in English; (3) participants were adults and/or children (we did not exclude articles on the basis of participant age) with a TBI of any severity from subconcussive through severe; and (4) DTI was performed at one or more time points. Exclusion criteria included: (1) articles in languages other than English; (2) studies conducted on animals or *in vitro*; (3) the primary disease focus was a disease other than TBI (including post-traumatic stress disorder, post-traumatic headache and tumors); (4) studies not employing DTI or advanced diffusion imaging; and (5) references that were not research studies (e.g., reviews, editorials, etc.) or that lacked full peer-review (e.g., conference abstracts, protocols, etc.). Due to the large volume of literature using DTI to study TBI from 2012 to 2022, this review will report only on those studies that focus on moderate and/or severe TBI. Those that focus on mild TBI will be discussed separately, in our companion paper, “DTI in Mild Traumatic Brain Injury- The Second Decade: A Systematic Review”.

### 2.4 Data extraction and quality assessment

References that passed the screening process underwent data extraction and quality assessment by two members of the review team using a customized form created in Covidence. The data extraction form collected information on the study and participant characteristics – such as study design, setting, participant demographics, injury severity, mechanism of TBI, and imaging details – along with the major outcomes. In addition, a quality assessment form drawing on selected questions from the quality assessment tools developed by the national heart, lung, and blood Institute^6^ was created in Covidence and used to evaluate each study.

## 3 Results

**Figure 1:**
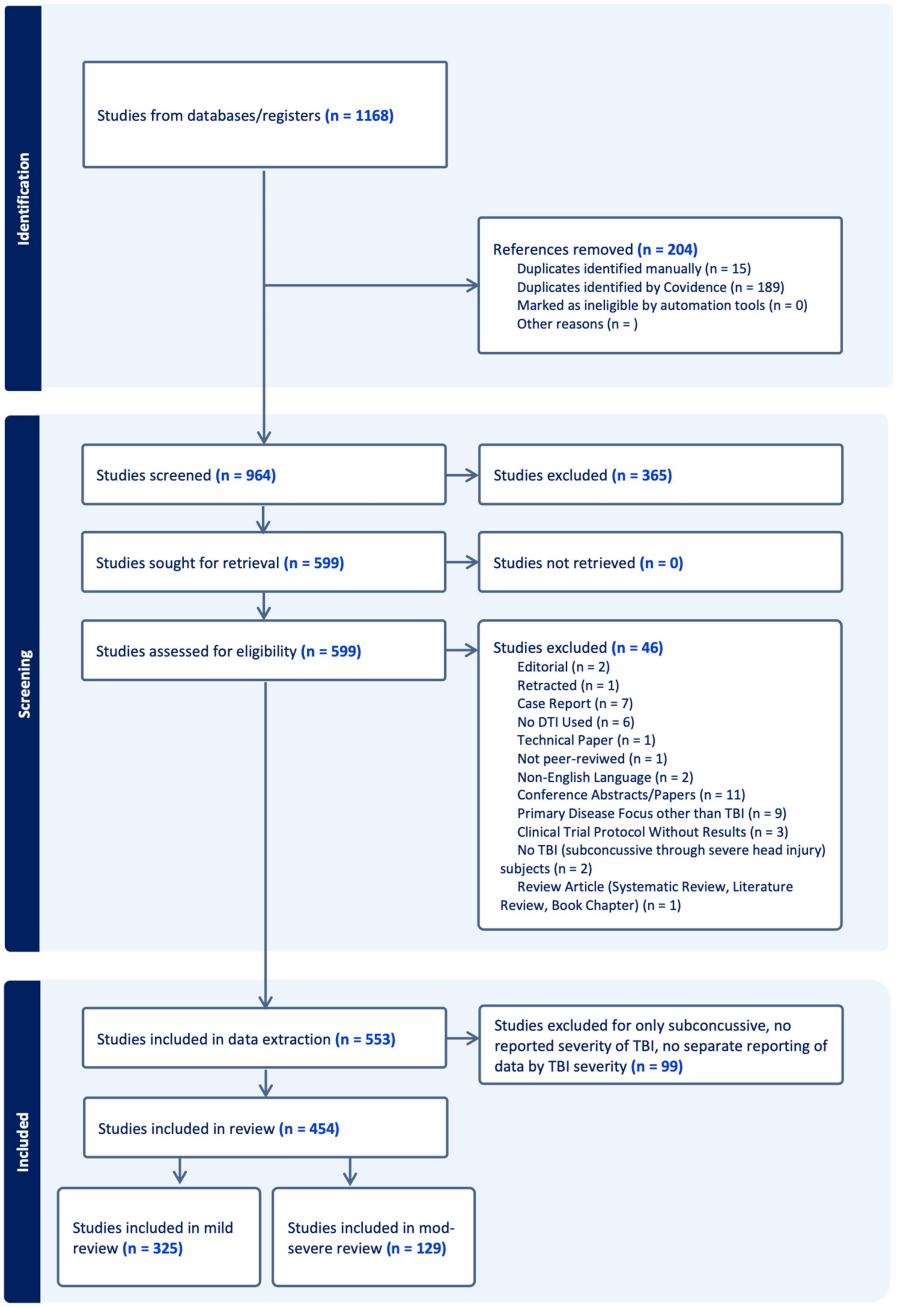
Preferred reporting items for systematic reviews and meta-analyses (PRISMA) flow diagram. Results of the initial search, title/abstract screening, and full text review, including reasons for exclusion are presented in the flow chart.

A total of 1,168 articles were imported into Covidence. After removal of 204 duplicates, 964 studies underwent title and abstract screening. 365 were excluded and full text was reviewed for the remaining 599 studies. Ultimately, 553 studies underwent data extraction and quality assessment. The PRISMA flow diagram is displayed in Figure 1.

The 553 articles included in the extraction phase of the systematic review were further filtered to exclude any studies that reported exclusively on subconcussive head impacts, did not report the TBI severity of study groups, or included participants with a range of TBI severities without reporting separately for severity subgroups (n = 99). Due to the large volume of studies, the articles were divided into two subgroups- mTBI only (n = 325) and msTBI (n = 129). Articles that included both mild and moderate–severe TBI patients, but reported findings separately for each category, were categorized according to the severity (mild or moderate–severe) of the majority of the participants. The two subgroups are reported in separate companion papers, with the present paper focused on the 129 studies addressing msTBI.

### 3.1 Publication frequency

**Figure 2:**
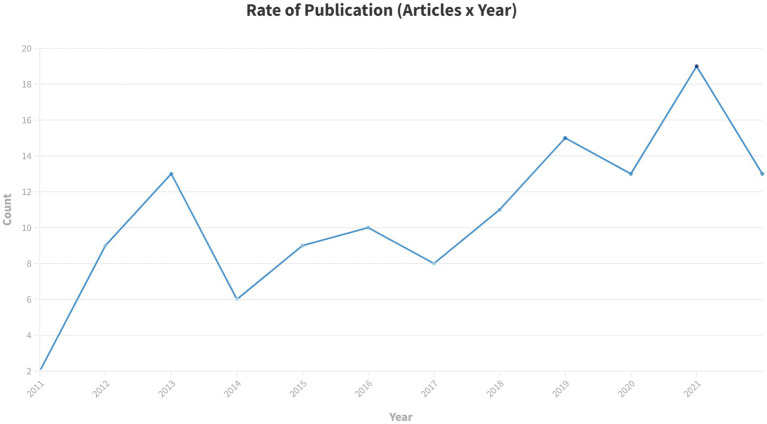
Rate of publication of studies that use DTI to study moderate–severe TBI from 2012 to 2022.

**Figure 3:**
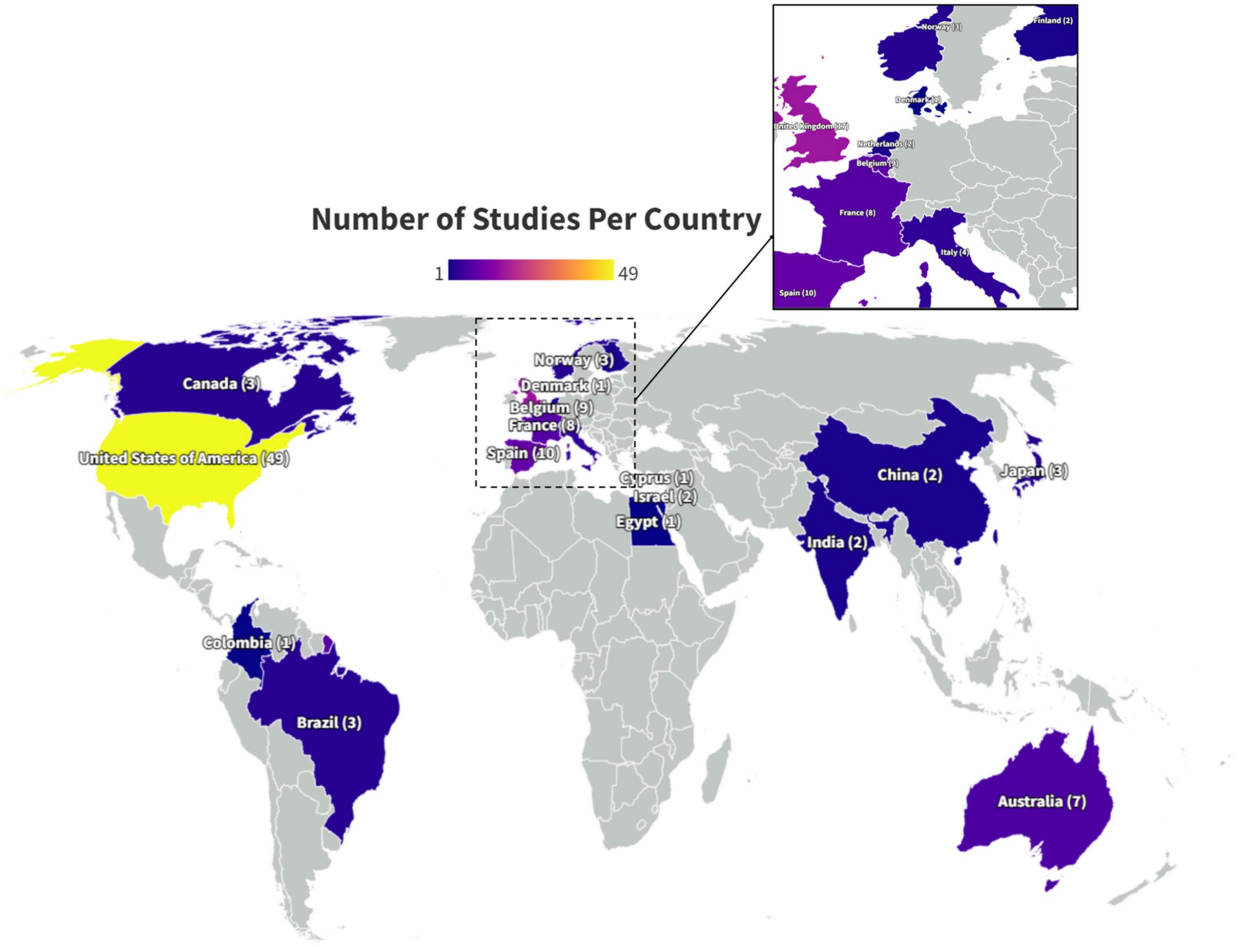
Geographic distribution of moderate–severe TBI studies. Country of origin, determined by where each study included in this systematic review took place, is denoted on the world map. The color of the country denotes how many papers took place in that country. The number of studies is included in parentheses. The fewest studies took place in countries colored dark blue, while the most numerous studies took place in countries colored lighter purple and yellow. Parts of the world that are colored gray, without a country name identifier or number in parentheses, did not conduct a study of DTI in msTBI that was included in this systematic review.

Over the past decade there has been an overall increase in the yearly publication rate with intermittent declines (Figure 2). The settings for papers studying moderate/severe TBI using DTI are geographically diverse (Figure 3). The studies included in this review were conducted in North and South America, Europe, Africa, Asia and Australia, with the greatest number of studies, 49/129 (38%) conducted in the US (8–56). The geographic distribution of studies is greater for studies of msTBI than for mTBI. This may be due to perceived clinical importance of more severe TBI worldwide and the hospital presentation that is typical in more severe injury.

### 3.2 TBI patient demographics

Demographic features of patients enrolled in msTBI studies are detailed in Table 1. More men (76.4%) than women were included as participants in the msTBI studies. This is consistent with epidemiologic cohort studies that have found that men experience msTBI at higher rates compared to women (57, 58). The variation in demographic features and mechanism of injury across studies limits integration of patient data and formulation of inferences aimed at specific features or mechanisms. While there were no specifically recruited athlete populations for msTBI, which differs from studies of mTBI where athletes are often specifically studied, 11.63% of participants were injured in the setting of athletics. It is also possible that some participants were included in more than one sample, as various studies published by the same authors reported similar patient sample characteristics. Eight pairs of studies may have overlapping participant enrollment (38, 48, 49, 54, 59–70).

**Table 1:** Overview of demographic data for included msTBI studies.

Demographic variables	Value
Study subjects	
Total msTBI participants	9,609
Average msTBI participants per study	74
Range of msTBI participants per study	8–246
Sex	
Male	76.40%
Female	23.60%
Age	
Average Age (years)	32.96
Age Range (years)	12–70
Number of Studies with patients <18 years old	23
Population studied	
General/Civilian	92.25%
Sports	0%
Military	4.65%
Unspecified	4.65%
Mechanism of injury	
MVA, Falls, Assaults	69.77%
Sports	11.63%
Military Blasts	3.1%
Mixed: Sports, MVA, Falls, Assaults	10.08%
Mixed: Blast, Sports, MVA, Falls, Assaults	1.55%
Not reported	28.68%

Number of patients, sex, age, population, and injury mechanism are described in the table. Blasts refer to msTBI sustained due to explosions in the military setting. Mixed mechanism of injury includes studies with patients who sustained msTBI due to a combination of motor vehicle accidents (MVA), falls, assaults, sports, or blasts.

### 3.3 Severity, chronicity, and study design

Some studies did not distinguish mTBI and msTBI in their reporting. Since these studies were excluded from the review, some msTBI patients are likely not included in our analysis. Severity of TBI was typically determined by Glasgow Coma Scale (GCS) score, with definitions of mild (GCS: 13–15), moderate (GCS: 9–12), and severe (GCS: 3–8) consistent across the studies. If a GCS score was not reported, severity reported by the authors was assumed to be accurate. Of the 129 studies that included either or both moderate and severe TBI, 98 reported on moderate TBI (8–15, 17, 20–25, 27, 29, 30, 32–38, 41, 42, 44–49, 51–55, 59–66, 69–120), and 125 reported on severe TBI (8–51, 53–56, 59–77, 79–85, 87–117, 119–138). Sixteen studies included mild with msTBI (8, 9, 13, 15, 20, 29, 30, 33, 34, 73, 74, 77, 84, 99, 101, 111). One study reported only mild and severe TBI, excluding moderate TBI patients (43).

**Figure 4:**
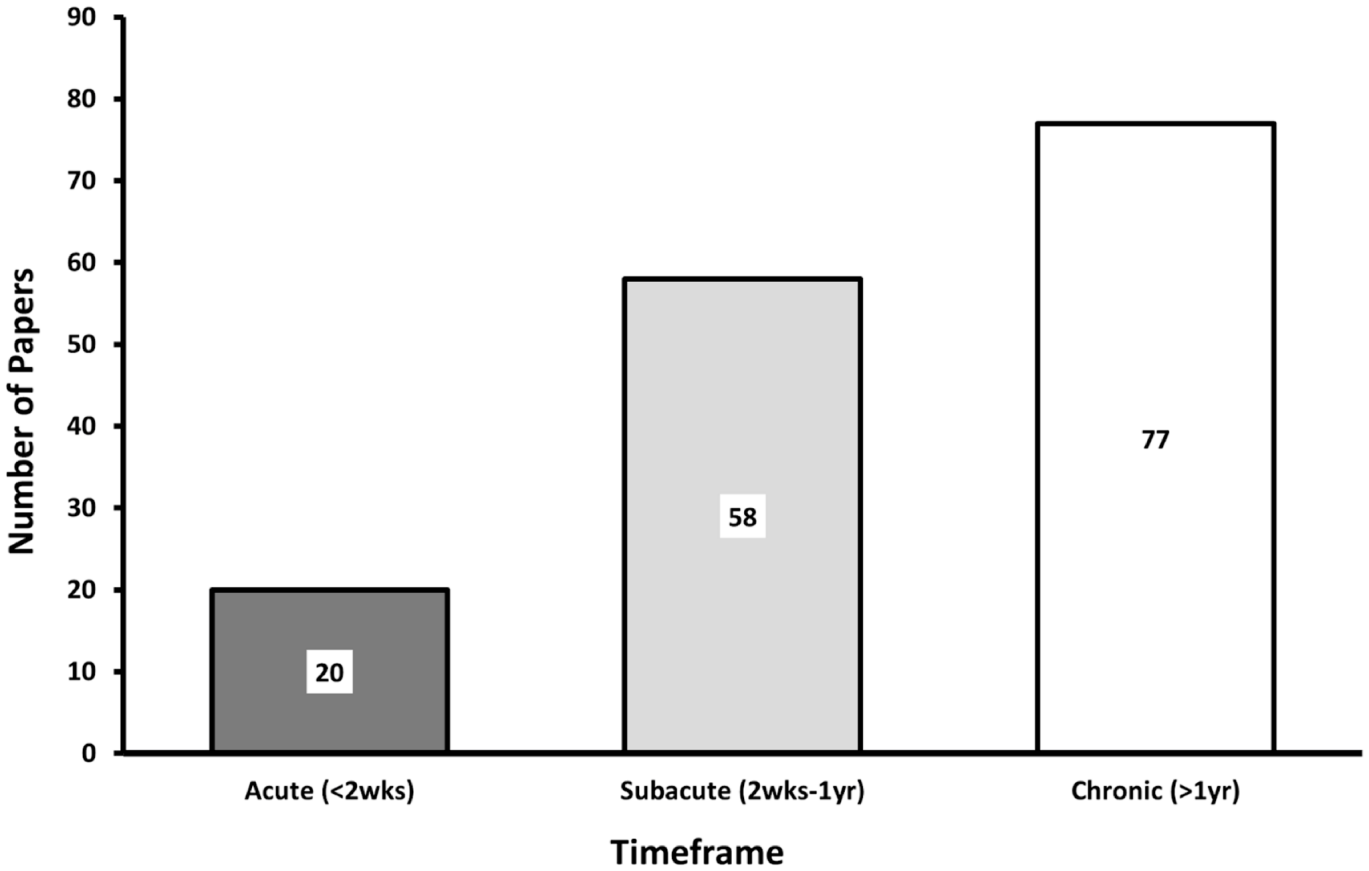
Post-injury DTI acquisition. This bar graph denotes when DTI was acquired in the included studies. Studies were only included if there was sufficient information to determine the chronicity of individual patient injuries. Studies may be included in more than one category if they studied patients at multiple timepoints. Thus, the total number of studies represented in this graph exceeds the total of included papers (acute: <2 weeks, Subacute: 2 weeks1 year, hronic: >1 year).

The timing of study assessments after TBI also varied across papers. We classified papers according to 3 timeframes following TBI: acute (<2 weeks), subacute (2 weeks - 1 year) and chronic (>1 year). Most msTBI papers reported on the chronic phase of injury (Figure 4), whereas studies of the subacute phase predominated in studies published 2002–2012 (6). The ability to predict recovery from msTBI remains a clinical conundrum that is of great interest to patients, families, and clinicians. Hence, this shift of focus to chronic msTBI may reflect an interest in better understanding brain abnormalities and prognosis following injury. Clinical outcomes associated with DTI measures are discussed later in this review (*Associations of DTI with Patient Outcomes*).

**Figure 5:**
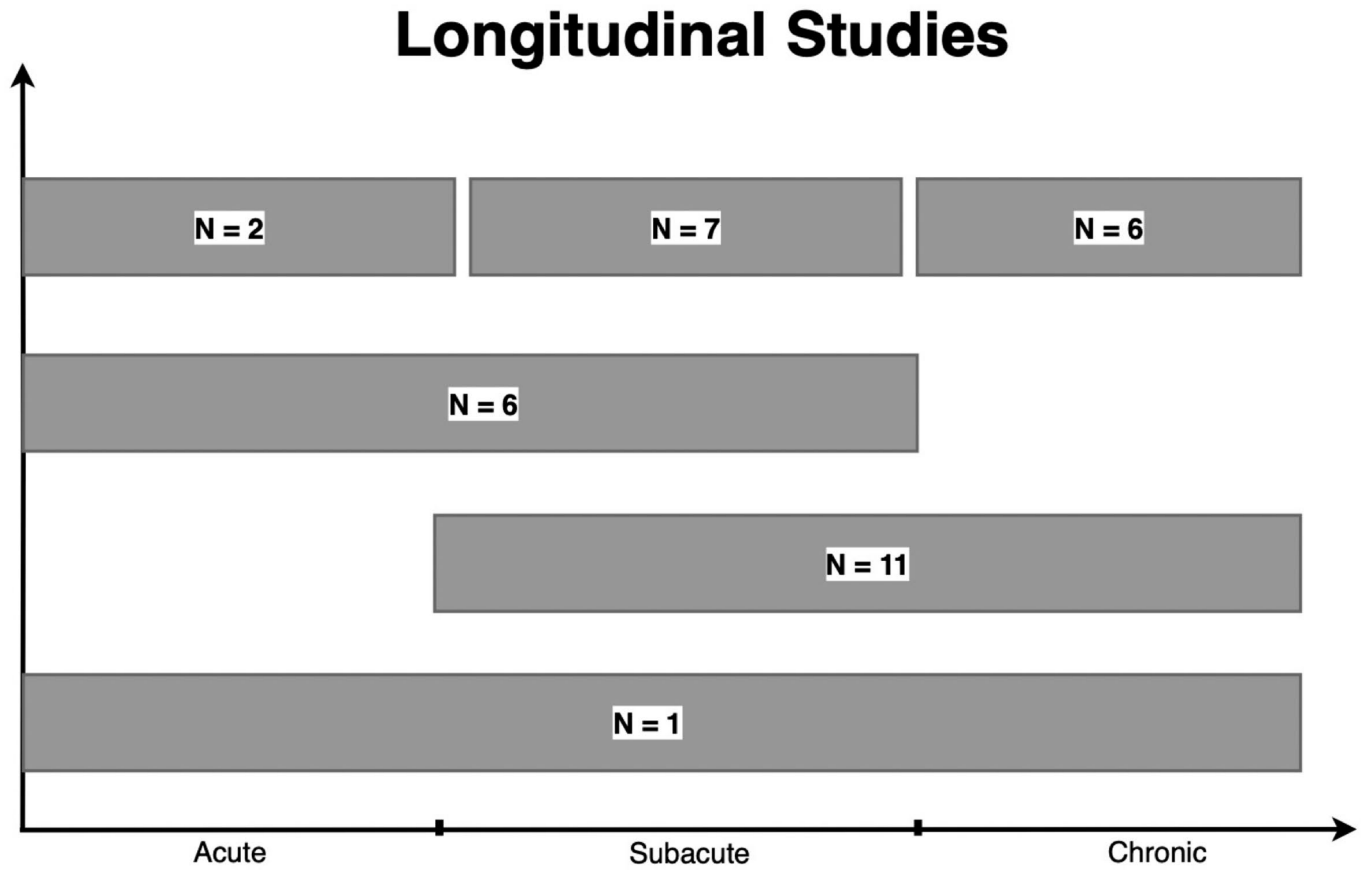
Longitudinal studies. A total of 33 studies reported longitudinal data. “*N*=” represent the number of studies within each grouping. One study examined patients in the acute, subacute, and chronic phases of TBI. Eleven studies examined patients in the subacute to chronic phase, while 6 studies examined patients within the acute to subacute phase. Finally, 2, 7, and 6 studies obtained a DTI scan on the same patient more than once in the acute, subacute, and chronic phases, respectively. *Baseline represents the number of studies including a scan prior to injury among longitudinal studies. This number is not added to the total of longitudinal studies. (Acute: <2 weeks, subacute: 2 weeks–1 year, chronic: >1 year).

Thirty Three of the 129 (25.6%) papers included in our review evaluated the same group of patients with DTI at multiple time points following injury (9–11, 18, 25, 33, 35–39, 41, 44, 52, 54, 59, 60, 63, 64, 69, 71, 76, 86, 88, 89, 93, 95, 105, 107, 119, 129, 134, 138). As shown in Figure 5, many of these studies were in the subacute-chronic phase post-injury. In comparison, the prior decade found only 13% of studies evaluated patients with DTI at multiple time points (6). Despite the logistical difficulty and costs associated with longitudinal DTI studies, they offer valuable insights into long-term changes in brain pathology and, consequently, patient prognosis.

Most studies utilized cohort study design (82.17%) (8–13, 16, 18–23, 25–30, 32–34, 36–38, 40–44, 46–51, 53–56, 59–62, 64–66, 68, 70, 71, 73–78, 82–84, 86–94, 97–100, 102–106, 108–126, 128, 129, 131–138), followed by case–control (6.98%) (14, 15, 17, 24, 67, 72, 85, 101, 127), randomized controlled (4.65%) (35, 39, 52, 79, 95, 107), cross sectional (4.65%) (31, 45, 80, 81, 96, 130), and before-after (1.55%) (63, 69) study designs. Our classification of study design aims to clarify how studies were conducted by including additional study design descriptors to capture the breadth of literature in this field. The majority of studies from 2002 to 2012 were described as cross-sectional, indicating a single time point at which participants with TBI were studied. Many studies identified msTBI patients in the ED or hospital setting and completed the DTI scan with or without additional clinical assessment at a later date. We considered this a cohort study design, while it would have been considered a cross-sectional design in the previous review. This difference in study design assignment prevents direct comparison of our analyses on the first and second decade. This approach, however, more precisely describes the study designs reported during the second decade. Cohort studies enroll participants with an exposure, in this case TBI, and assess an outcome, in this case DTI or a clinical outcome such as cognitive performance, at a later date. Cohort studies could measure outcomes once and at multiple time points. Cross sectional studies provide a “snapshot” of exposure and outcome at a single moment in time. In msTBI where patients are often hospitalized and require intensive medical and neurologic care during the acute timeframe, outcomes are often measured in the post-acute period.

Control groups were included in 85.27% of studies (8–17, 19–27, 29–34, 36–38, 41, 44, 47–51, 53–56, 59–62, 64–78, 80–89, 91–102, 104–116, 118, 122, 123, 125–138), while 14.73% of studies did not include controls (18, 28, 35, 39, 40, 42, 43, 45, 46, 52, 63, 79, 90, 103, 117, 119–121, 124). One hundred two studies enrolled healthy controls (8–17, 19, 22–26, 31–34, 36–38, 41, 44, 48–51, 53–56, 59–62, 64–67, 69–78, 80–89, 91–98, 100–102, 104–116, 118, 122, 123, 125–138), 9 enrolled orthopedic injury controls (9, 10, 14, 20, 21, 29, 47, 74, 99), and 3 enrolled military controls (27, 29, 30). Studies that did not include a non-TBI control group investigated the association of DTI measures with clinical outcomes among patients with msTBI or stratified the msTBI group by injury, imaging, or outcome features to compare subgroups. Subgroups were based on the presence of symptoms such as weakness (118), comorbid PTSD (27), or chronicity of injury (37).

### 3.4 Data acquisition parameters

One hundred three studies utilized MR scanners with magnetic field strength of 3.0 T (8, 9, 11–14, 16–20, 22–34, 36–42, 44, 45, 47–56, 63–79, 82–85, 87–89, 91, 92, 94–102, 104–109, 113–117, 120, 122, 123, 125–130, 132, 133, 135, 137, 138), 28 studies used 1.5 T (10, 15, 21, 35, 43, 46, 54, 59–62, 86, 90, 93, 103, 110–112, 118, 119, 121, 123–125, 131, 132, 134, 136), and 2 studies did not report magnetic field strength (80, 81). While there was approximately equal use of 1.5 T and 3.0 T scanners in the previous decade (6), 3.0 T MRI scanners predominate in more recent DTI studies of msTBI, likely due to the increasing availability of higher field strength in clinical and research settings. Greater magnetic field strength provides enhanced signal to noise ratio (SNR), which can be leveraged to shorten acquisition time while enhancing spatial resolution and/or increase the number of diffusion-sensitizing directions, potentially allowing smaller, more subtle microstructural alterations to be detected (139). Although higher magnetic field strength scanners, such as 7.0 T, have become more widely available, no study of msTBI employed field strength greater than 3.0 T.

The b-value is a parameter that reflects the strength and timing of the diffusion-sensitizing gradient magnetic fields, with higher b-values resulting in greater diffusion-related signal effects, but lower SNR (140). Five articles did not report the b-values employed (39, 80, 82, 99, 125). Of the studies that reported b-values, 116 were single-shell studies (using one unique non-zero b-value), with a b-value ranging from 700 s/mm2 to 3,000 s/mm2 (8–15, 17–34, 36–38, 40–51, 53–56, 59–79, 81, 83–87, 90–92, 94–98, 100–115, 117–122, 124–138). There were 8 multi-shell studies (using several unique non-zero b-values) with b-values ranging from 50 s/mm2 to 3,000 s/mm2 (16, 35, 52, 88, 89, 93, 116, 123). Of these multi-shell studies, 1 study used two b-values (16), 3 studies used three b-values (35, 93, 123), 1 study used four b-values (116), 1 study used five b-values (52), and 2 studies used 6 b-values (88, 89). While the majority of studies used a single b-value, multi-shell techniques, which offer potential to more precisely characterize the nature of water diffusion in tissue and its alteration by microstructural features, are gaining popularity, as evidenced by the increasing number of studies employing advanced diffusion methodologies compared to 2002–2012 (6).

The reported number of diffusion-sensitizing directions across studies ranged from 10 to 96 with an average of 36 and a mode of 30 diffusion-sensitizing directions. Eight studies did not report the number of diffusion-sensitizing directions (36, 46, 48, 49, 80, 82, 90, 93). Increasing the number of diffusion-sensitizing directions can increase the accuracy of diffusion scalar and diffusion direction estimates, but at the cost of additional image acquisition time (140, 141). The number of diffusion-sensitizing directions used in included studies has increased compared to the previous decade, in which the range was 6 to 64 with an average value of 27 (6).

**Figure 6:**
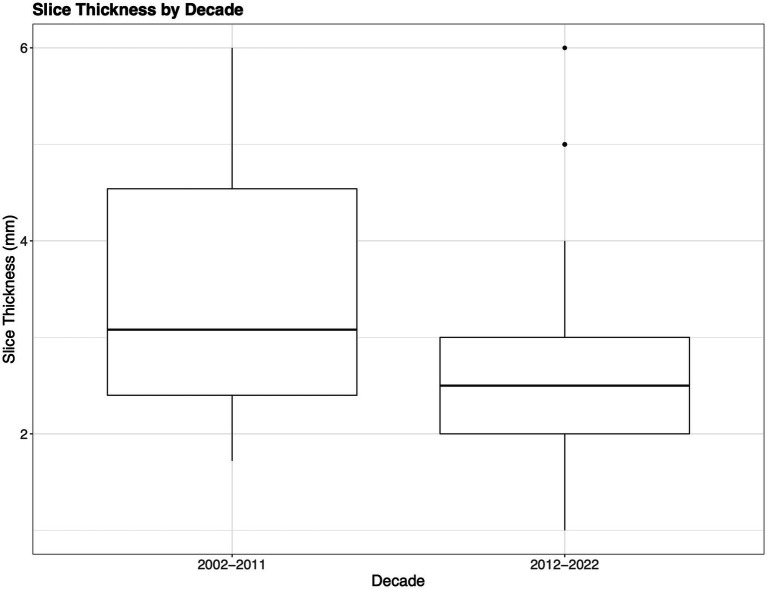
Slice thickness. The boxplots show the difference in acquisition slice thickness from the initial decade compared to the most recent decade. Notably decade 1 (2002–2011) includes mild–severe TBI, while decade 2 (2012–2022) only indicates slice thickness for moderate–severe TBI.

With respect to slice thickness, the mean reported value was 2.76 mm (median 2.5 mm, range 1.0–6.0 mm, mode 2.00 mm), with 24 articles not reporting slice thickness (9, 12, 23, 36, 39, 45, 48, 49, 52, 54, 55, 70, 75, 79, 80, 82, 83, 85, 89, 99, 117, 121, 133, 137). As the slice thickness decreases, the axial resolution of the images increases and SNR decreases (140). Studies during the past decade have used, on average, a somewhat thinner slice compared to 2002–2012, affording investigators better axial resolution, which is demonstrated in Figure 6 (2.76 mm compared to 3.08 mm) (6).

When evaluating the use of DTI across studies, it is important to consider the different imaging parameters utilized, including strength of the magnetic field, choice and number of b-values, number of diffusion-sensitizing directions, and choice of slice thickness. Choice of imaging parameters impacts sensitivity of the diffusion measures to detect tissue alterations and can determine the potential to define more detailed features using advanced models such as neurite orientation dispersion density imaging (NODDI). NODDI uses multiple b-values to model the contributions of water diffusion within the extracellular, intracellular, and CSF compartments. Understanding the variation in parameters employed across studies is important for the interpretation of results, assessment of study conclusions, as well as for advancing the field toward standardized and optimized imaging protocols. While there has been increasing emphasis on standardization and reporting of acquisition parameters, we did not observe that studies have converged on common parameters for b-values, slice thickness, or diffusion directions. Despite differences in acquisition parameters, data harmonization across sites and studies is advancing. Initiatives such as the ENIGMA consortium have created protocols to allow for harmonization across sites and scanners in order to create large datasets with greater power for detecting differences and for use in genomics studies (142, 143).

### 3.5 Data analysis methods

msTBI studies used either a region of interest (ROI) or whole-brain approaches. ROI were either atlas-derived or manually delineated. Twenty-eight studies employed manual ROI delineation (14, 19, 21, 23, 28, 30, 31, 39, 40, 43, 46, 61, 62, 64, 68, 77, 86, 88, 91, 96, 101, 104, 106, 110, 118, 120, 121, 133). Of these, only 6 reported reliability testing of ROI placement (19, 21, 31, 61, 88, 101). Sixty-one studies employed atlas-derived ROIs (8, 9, 11–13, 17, 18, 20, 22, 26, 29, 32, 38, 40, 45, 47, 48, 50, 53, 54, 59, 60, 65, 71, 74–76, 78, 79, 81, 82, 84, 85, 87, 89, 93, 94, 98–100, 102, 103, 105, 107–109, 111–115, 119, 120, 123, 126, 129, 132, 134–137). Of the 68 studies using whole-brain analysis, 66 studies used voxelwise approaches (8–10, 12, 13, 15, 16, 22, 23, 25, 27, 30, 33, 34, 37–39, 41, 42, 44, 48, 49, 51–53, 55, 56, 63, 65–67, 70, 72, 73, 77, 80, 81, 83, 89, 90, 92, 94, 97–100, 102–104, 106, 116, 117, 119, 120, 122, 124, 127, 128, 130–136, 138), and 2 used whole brain, hemispheric, and ROI histogram analysis (24, 92). Four studies used other analysis approaches such as fixel-based analysis and an automated multi-atlas tract extraction (36, 53, 69, 95). Thirty-two studies used a combination of ROI and whole-brain analysis (8, 9, 12, 13, 17, 22, 23, 30, 38, 39, 48, 53, 65, 77, 81, 89, 92, 94, 98–100, 102–104, 106, 119, 120, 132–136). Single subject voxelwise analysis, where abnormal regions are determined separately in each patient, was used in 8.53% of the included msTBI studies (10, 46, 48, 50, 70, 80, 85, 111, 116, 129, 138).

The ROI analysis method entails *a priori* specification of a region or WM tract of interest, from which diffusion scalar measures are extracted for further analysis (140). The ROI approach allows for hypothesis-driven testing of selected brain areas, perhaps on the basis of functional- or injury mechanism-related factors (140). This approach can be particularly relevant when investigators test for associations of WM microstructure in a specific region with cognitive or behavioral symptoms expected from injury to that brain region. Whole-brain approaches initially consider all brain voxels and identify abnormal diffusion measures in each voxel regardless of region (140). Voxel-based approaches are automated, provide greater spatial resolution, and do not require *a priori* ROI selection (140). Single subject analysis methods identify abnormalities on a per-subject basis by comparing the individual of interest to a group of controls.

Compared to 2002–2012, a greater proportion of papers in the most recent decade reported voxelwise/Tract-Based Spatial Statistics (TBSS) whole-brain analyses (50.1%) compared to the previous decade (17.0%) (6). However, more studies continue to use ROI analyses compared to voxel-wise analyses, with 19.5% of studies using both ROI and voxelwise methods. Approximately the same proportion of ROI studies (73.9%) and voxel-wise studies (72.7%) reported significant differences in DTI parameters between the groups studied. Even when both ROI and voxel-wise analyses were used within the same study, approximately the same proportion (73.3%) of studies found significant differences between the msTBI group and the control group. Notwithstanding advantages and disadvantages of each analysis method, this review indicates they are similarly likely to detect significant group differences in msTBI.

### 3.6 Diffusion measures studied

Diffusion MRI is performed to facilitate modeling the diffusion-weighted MRI signal from each image voxel to generate quantitative metrics, including measurements of the degree of anisotropy and dominant direction of diffusion (140). In the DTI model, the diffusion process can be represented as a 3D ellipsoid defined by three vectors (λ1, λ2, λ3). These three vectors can be used to compute scalar summary measurements at each voxel, which include but are not limited to fractional anisotropy (FA), a measure of directional coherence of water; mean diffusivity (MD, also referred to as the apparent diffusion coefficient (ADC)), a measure of total direction-independent diffusion; radial diffusivity (RD), a measure of average diffusion along 2 minor axes of the diffusion ellipsoid; and axial diffusivity (AD), a measure of diffusion along the principal axis of the diffusion ellipsoid (144).

FA was the most commonly studied DTI scalar measurement across all articles (87.6%) (8–16, 18–31, 33, 34, 36–45, 47–49, 51–54, 56, 59–69, 71–78, 80–84, 86, 87, 89–92, 94, 96–107, 109–113, 116–130, 132–138). MD/ADC was the second-most common DTI scalar measurement studied (54.26%) (9–11, 16, 19–24, 26, 27, 29, 30, 31, 34–40, 43, 44, 46–49, 51–54, 63, 64, 67–69, 71, 72, 74–76, 78, 83, 86–89, 91–93, 99, 101, 104, 107, 109, 116, 121, 123–125, 128–130, 132, 134–138) and less commonly studied were RD (28.68%) (9, 14, 22, 24, 26, 27, 29, 30, 33, 36–39, 43, 44, 51, 52, 54, 55, 59, 60, 63, 64, 72, 73, 75, 83, 89, 91, 99, 123, 129, 132–134, 136, 138) and AD (27.13%) (9, 14, 22, 24, 26, 27, 29, 30, 33, 36–38, 43, 44, 51, 52, 54, 59, 60, 63, 64, 72, 73, 75, 83, 89, 91, 99, 123, 129, 132–134, 136, 138). Of the studies that analyzed FA in msTBI, 77/112 (68.8%) reported significant group-wise differences (8–12, 14–16, 19–23, 26, 27, 29, 31, 33, 34, 36, 38, 41, 44, 47–49, 53–55, 59–62, 64–69, 71–75, 77, 78, 80–83, 86, 87, 89, 91, 96, 97, 100–102, 104–107, 109–112, 116, 118, 122, 123, 126, 128, 129, 134, 135, 137). All but one of the 77 (98.7%) studies found lower FA in msTBI compared to the controls. That one study found some brain regions with lower FA and others with higher FA in the TBI group compared to controls (10). The vast majority (36/39) of studies reporting on MD found higher MD in the msTBI group (9, 11, 22, 26, 27, 29, 36–38, 47–49, 51, 53, 54, 64, 67–69, 71, 72, 74, 78, 83, 86, 87, 91, 101, 104, 107, 109, 116, 123, 134, 135, 137). Similarly, 19/20 studies reporting on RD found higher RD in the msTBI group compared to controls (14, 26, 27, 29, 33, 36–38, 44, 51, 54, 55, 59, 72, 73, 83, 91, 107, 123). Findings of studies reporting on AD were mixed with 10/17 studies reporting higher AD in the msTBI group (9, 14, 22, 27, 29, 51, 64, 83, 123, 134) and 5/17 studies reported lower AD (33, 54, 59, 60, 75). In two of the 17 studies, the direction of the group difference was not consistent across all ROIs (44, 91). Rather than a uniform pattern across the brain, patients with TBI showed both increases and decreases in AD compared to controls depending on the region being examined. Lower FA is thought to reflect loss of microstructure elements, such as myelin and axons, as well as glial proliferation (145). These pathologic features similarly lead to elevation of MD, RD, and ADC, due to less restriction of water diffusion within injured tissue (140).

The brain region most commonly found to exhibit significant differences of DTI measures compared to controls was the corpus callosum, with 47 articles reporting significant findings in the corpus callosum among 94 articles that found significant groupwise differences (8, 10, 14, 15, 20, 23, 26, 27, 31, 33, 36–38, 44, 53, 54, 56, 59, 60, 62, 64, 65, 67–69, 71, 73, 74, 77–79, 82, 86, 89, 91, 94, 105, 110–112, 116, 118, 122, 128, 129, 133, 134). Other commonly reported regions included the thalamic radiation, cingulum, inferior fronto-occipital fasciculus, longitudinal fasciculus, and internal capsule. Indices of less restricted diffusion such as lower FA, higher MD, and higher RD in these white matter tracts is consistent with axonal damage and/or demyelination. While these findings are compatible with the proposed pathophysiology of traumatic axonal injury in patients with msTBI, it is important to note that the heterogeneity of planned analyses, reported parameters as well as the white matter tracts evaluated is a potential limitation of the literature and source of selection and publication bias.

### 3.7 Advanced diffusion techniques studied

Advanced diffusion imaging approaches, such as diffusion-based connectivity, diffusional kurtosis imaging (DKI), and NODDI have been less widely reported in msTBI and are not the main focus of this review. 11.63% of studies (15/129) reported results from advanced neuroimaging techniques (16, 55, 70, 84, 85, 94, 95, 106, 108, 113–116, 126, 136). Diffusion-based connectivity analysis was the most widely used advanced diffusion method (70, 84, 94, 106, 108, 113–115, 126, 136). Diffusion-based connectivity maps neuronal structural connections across brain networks to provide insight into the integrity of structural brain networks and how they are altered by injury. DKI, which characterizes non-gaussian diffusion behavior more accurately than DTI, and NODDI, which models diffusion within the intracellular, extracellular and free water compartments to provide a more precise biophysical characterization of tissue water diffusion, were each reported in a single msTBI study (16, 116). Fewer studies of msTBI (11.63%) employed advanced diffusion techniques compared to mTBI (20.7%) during this decade. These advanced methods were first described at the end of the first decade of reported use of DTI in TBI and therefore their use was not described in the initial review (6). Given that there is less variability in DTI results in msTBI compared to mTBI, there has likely been less interest in employing advanced diffusion methods. Over time as comfort with these new techniques grows, implementation is likely to rise to increase as well.

### 3.8 Associations of DTI with patient outcomes

Many studies went beyond group comparisons of DTI measures between patients and controls and evaluated associations of DTI measures with msTBI outcomes. Thirty studies investigated associations between clinical outcomes and DTI measures (11, 17, 20, 23, 26, 43, 47, 54, 55, 59, 60, 62, 63, 65, 71, 73, 83, 90, 91, 96, 104, 107, 112, 115, 123, 130, 133, 135, 136, 138). Thirty six studies investigated associations between cognitive tasks and DTI measure (12, 14–16, 19, 22, 27, 29, 31–33, 37, 41, 42, 45, 51, 53, 64, 67–69, 71, 72, 74, 77, 79, 86, 87, 89, 97, 109, 116, 117, 122, 127, 134). There was variability in the timing of patient outcome assessments relative to DTI acquisition. Measures like GCS and post-traumatic amnesia were obtained close to the time of injury. Cognition, global outcomes, and mood/behavior symptoms were often assessed more remote from injury (weeks-years). As above, the most common timing of DTI acquisition was in the chronic phase of injury (>1 year). Table 2 summarizes studies that investigated associations of the most common clinical measures with FA or MD. Clinical measures evaluated included global outcome measures such as the Glasgow Outcome Scale-Extended (GOS-E) and Coma Recovery Scale-Revised (CRS-R). More specific outcome domains included mood (e.g., anxiety, depression), balance, behavior and communication, and symptoms scores. GCS and post-traumatic amnesia represent clinical scales that characterize injury severity. Individual assessment tools varied among studies, however grouping these outcomes by domains allowed us to summarize study findings and draw conclusions despite the heterogeneous range of reported outcome measures. Global outcomes and mood symptoms were the most commonly studied clinical outcome measures. The majority of studies did not find a significant association of FA or MD with global outcome measures or with mood symptoms for msTBI patients. For the 11 studies reporting an association of DTI with global outcomes, the most consistent was for GCS, with higher GCS (less severe injury) associated with higher FA and lower MD (Table 2).

**Table 2:** DTI associations with clinical measures.

DTI measure	Association	Global outcome measures	GCS *Higher GCS = Less severe injury*	Mood symptoms	Balance	Behavior/Communication	PTA
FA	Positive	*Better outcome, Higher FA* 2(104, 112)	*Less severe injury, Higher FA* 5(34, 54, 62, 83, 104)		*Better balance, Higher FA*	*Worse communication, Higher FA*	*Longer amnesia, Higher FA*
	Negative	*Better outcome, Lower FA*	*Less severe injury, Lower FA*		*Better balance, Lower FA* 1(107)	*Worse communication, Lower FA* 2(23, 54)	*Longer amnesia, Lower FA* 2(55, 104)
	None	6(11, 26, 63, 65, 130, 133)	2(43, 91)	4(20, 34, 47, 71)		1(130)	1(83)
MD	Positive	*Better outcome, Higher MD* 1(130)	*Less severe injury, Higher MD*	*More symptoms, Higher MD* 1(47)	*Better balance, Higher MD* 1(107)	*Worse communication, Higher MD* 1(130)	*Longer amnesia, Higher MD* 1(104)
	Negative	*Better outcome, Lower MD* 1(104)	*Less severe injury, Lower MD* 4(34, 83, 91, 104)	*More symptoms, Lower MD* 1(20)	*Better balance, Lower MD*	*Worse communication, Lower MD*	*Longer amnesia, Lower MD*
	None	1(26)		2(34, 71)			1(83)

This table reports studies that tested associations between FA or MD and clinical outcome measures. Higher scores on global outcome measures (Glasgow outcome scale extended [GOS-E], command following, coma recovery scale–revised [CRS-R]) denote better outcomes. For the Glasgow coma scale (GCS), higher scores represent better neurologic function. For the single study of balance, higher balance scores signified better balance. For mood (anxiety, depression, PTSD, apathy, resilience), behavior, communication, and post-traumatic amnesia (PTA), higher scores represent worse symptoms.

Table 3 summarizes analyses of cognitive performance associations with FA or MD. Individual tests of cognition were grouped by domain to better understand the trends, as a variety of individual tests were used across the various studies. The strongest evidence from studies of cognitive function is for the association of FA with psychomotor speed, including tests of simple reaction time and more complex psychomotor functions. Thirteen out of 18 studies found that lower FA was associated with poorer psychomotor or processing speed. The results for the remaining domains were more mixed. 7/13 studies found that lower FA was associated with poorer memory, with 5 out of the remaining 6 studies finding no association and one study finding lower FA associated with better memory performance. Half of studies that investigated the association between FA and composite cognition scores, reported lower FA was associated with poorer overall cognitive performance. Half of the studies that investigated language (reading, verbal fluency) also found that lower FA was associated with poorer language performance. MD was less commonly investigated, but across domains, when a significant association was identified, higher MD was associated with poorer cognitive function. For overall cognition, attention, executive function, memory, psychomotor speed, IQ, and language, about half of studies in each domain found a significant association of higher MD with poorer cognitive performance, while the other half found no association. A single study reported an association between higher MD and better executive function and psychomotor speed.

**Table 3:** DTI associations with cognitive measures higher scores in each of the domains is associated with better performance.

DTI measure	Association	Overall cognition	Attention	Executive function	Memory	Psychomotor/Processing Speed	Visuospatial	IQ	Verbal Fluency/Language Tasks/Reading Fluency
FA	Positive *Poorer performance, Lower FA*	4 (16, 27, 53, 90)	1 (64)	3 (19, 41, 109)	7 (14, 29, 37, 39, 64, 72, 127)	13 (12, 29, 31, 33, 37, 39, 41, 45, 69, 77, 89, 109, 122)		1 (64)	4 (27, 39, 73, 77)
	Negative *Poorer Performance, Higher FA*	1 (27)		2 (11, 104)	1 (15)	1 (104)			2 (15, 77)
	None	3 (29, 73, 77)	3 (73, 77, 87)	2 (29, 73)	5 (68, 73, 77, 87, 122)	4 (11, 55, 68, 87)	1 (14)	2 (87, 122)	2 (64, 122)
MD	Positive *Poorer Performance, Lower MD*			1 (104)		1 (104)			
	Negative *Poorer Performance, Higher MD*	2 (16, 27)	1 (64)	2 (11, 109)	4(29, 64, 69, 72)	4 (29, 37, 89, 109)		1 (64)	2 (27, 64)
	None	1 (53)	1 (87)	1 (29)	4(37, 39, 68, 87)	3 (39, 68, 87)		1 (87)	1 (39)

For each domain, a positive correlation indicates that lower FA or MD is associated with poorer performance, whereas a negative association indicates that lower FA or MD is associated with better performance.

### 3.9 Risk of Bias assessment

Structured risk of bias assessment was completed for each study included in the review, with questions depending on the study design. For the 123 studies categorized as cohort, case–control, or cross-sectional studies, 100% clearly stated the research question or objective. 92.7% (110/123) clearly specified and defined the study population. 89.4% (110/123) of studies selected or recruited subjects from the same or similar populations throughout the study. 99.2% (122/123) of studies clearly defined and implemented valid and reliable exposure measures across study participants. 86.2% (106/123) of studies identified key potential confounding variables and adjusted statistically for their impact. Thus, the overwhelming majority of studies adhered to essential principles of study quality. Identification of and adjustment for confounding variables was the most notable pitfall.

### 3.10 Limitations

Our review must be considered in the light of several limitations. We have limited our search to English-language peer reviewed original research articles. This search strategy therefore does not encompass gray literature (conference papers, abstracts, etc.) and papers published in languages other than English. However, given our broad search criteria, we believe our search results comprehensively capture the landscape of peer-reviewed literature on DTI in TBI over the decade 2012–2022. We cannot exclude publication bias toward those studies that reported positive results and had larger sample sizes, which are more likely to be submitted and accepted for publication. In classifying the studies by TBI severity, those that did not specify TBI severity or included more than one TBI severity without specifying the sample breakdown by severity were excluded from the review. However, only 11 of the 553 articles did not report TBI severity. These exclusions, therefore, are unlikely to bias our conclusions regarding the msTBI literature over the past decade. Since eight pairs of studies may have overlapping participant enrollment (38, 48, 49, 54, 59–70), some findings may be disproportionately represented. We did not exclude potentially overlapping participants. However, given the limited number of overlapping studies, this is unlikely to significantly impact our overall findings. Finally, due to the broad scope of this review, heterogeneity across studies with respect to factors such as design, acquisition and analysis methods, and result reporting preclude a more quantitative analysis of this literature such as meta-analysis.

## 4 Conclusion

Since its first decade (2002–2012) of reported use, DTI applications to msTBI have continued to expand in both quantity and scope, including notable increases in the proportions of larger and longitudinal studies, those employing whole brain analyses and those addressing clinical outcomes. The most salient finding across studies remains similar to 2002–2012, that despite heterogeneity of clinical and technical features of the individual studies, lower FA is consistently identified in msTBI patients compared to controls. While there are advantages and disadvantages of analysis methods, whole brain and region of interest approaches had approximately the same rate of significant groupwise findings. Further standardization of reporting and methods for data harmonization hold potential for the pursuit of larger “meta-studies,” with potential to confirm and advance knowledge beyond the power of individual cohorts.

## Data Availability

The original contributions presented in the study are included in the article/Supplementary material, further inquiries can be directed to the corresponding author/s.

## References

[1] Dewan MC Rattani A Gupta S Baticulon RE Hung Y-C Punchak M . Estimating the global incidence of traumatic brain injury. J Neurosurg. (2019) 130:1080–97. doi: 10.3171/2017.10.JNS17352, 29701556

[2] Centers for Disease Control and Prevention. CDC grand rounds: reducing severe traumatic brain injury in the United States. MMWR Morb Mortal Wkly Rep. (2013) 62:549–52. 23842444 PMC4604943

[3] Kaplan ZLR Van Der Vlegel M Van Dijck JTJM Pisică D Van Leeuwen N Lingsma HF . Intramural healthcare consumption and costs after traumatic brain injury: a collaborative European NeuroTrauma effectiveness research in traumatic brain injury (CENTER-TBI) study. J Neurotrauma. (2023) 40:2126–45. doi: 10.1089/neu.2022.0429, 37212277 PMC10541942

[4] Douglas DB Muldermans JL Wintermark M. Neuroimaging of brain trauma. Curr Opin Neurol. (2018) 31:362–70. doi: 10.1097/WCO.0000000000000567, 29878909

[5] Shenton ME Hamoda HM Schneiderman JS Bouix S Pasternak O Rathi Y . A review of magnetic resonance imaging and diffusion tensor imaging findings in mild traumatic brain injury. Brain Imaging Behav. (2012) 6:137–92. doi: 10.1007/s11682-012-9156-5, 22438191 PMC3803157

[6] Hulkower MB Poliak DB Rosenbaum SB Zimmerman ME Lipton ML. A decade of DTI in traumatic brain injury: 10 years and 100 articles later. AJNR Am J Neuroradiol. (2013) 34:2064–74. doi: 10.3174/ajnr.a3395 23306011 PMC7964847

[7] Page MJ McKenzie JE Bossuyt PM Boutron I Hoffmann TC Mulrow CD . The PRISMA 2020 statement: an updated guideline for reporting systematic reviews. Int J Surg. (2021) 88:105906. doi: 10.1136/bmj.n71, 33789826

[8] Zane KL Gfeller JD Roskos PT Stout J Buchanan TW Malone TM . Diffusion tensor imaging findings and neuropsychological performance in adults with TBI across the spectrum of severity in the chronic-phase. Brain Inj. (2021) 35:536–46. doi: 10.1080/02699052.2021.1887521, 33593218

[9] Yeh PH Lippa SM Brickell TA Ollinger J French LM Lange RT. Longitudinal changes of white matter microstructure following traumatic brain injury in U.S. military service members. Brain Commun. (2022) 4:fcac132. doi: 10.1093/braincomms/fcac132, 35702733 PMC9185378

[10] Wilde EA Ayoub KW Bigler ED Chu ZD Hunter JV Wu TC . Diffusion tensor imaging in moderate-to-severe pediatric traumatic brain injury: changes within an 18 month post-injury interval. Brain Imaging Behav. (2012) 6:404–16. doi: 10.1007/s11682-012-9150-y, 22399284

[11] Vijayakumari AA Parker D Osmanlioglu Y Alappatt JA Whyte J Diaz-Arrastia R . Free water volume fraction: an imaging biomarker to characterize moderate-to-severe traumatic brain injury. J Neurotrauma. (2021) 38:2698–705. doi: 10.1089/neu.2021.0057, 33913750 PMC8590145

[12] Ware JB Hart T Whyte J Rabinowitz A Detre JA Kim J. Inter-subject variability of axonal injury in diffuse traumatic brain injury. J Neurotrauma. (2017) 34:2243–53. doi: 10.1089/neu.2016.4817, 28314375 PMC5510712

[13] Vaughn KA DeMaster D Kook JH Vannucci M Ewing-Cobbs L. Effective connectivity in the default mode network after paediatric traumatic brain injury. Eur J Neurosci. (2022) 55:318–36. doi: 10.1111/ejn.15546, 34841600 PMC9198945

[14] Treble A Hasan KM Iftikhar A Stuebing KK Kramer LA Cox CS Jr . Working memory and corpus callosum microstructural integrity after pediatric traumatic brain injury: a diffusion tensor tractography study. J Neurotrauma. (2013) 30:1609–19. doi: 10.1089/neu.2013.2934, 23627735 PMC3787334

[15] Strangman GE O'Neil-Pirozzi TM Supelana C Goldstein R Katz DI Glenn MB. Fractional anisotropy helps predicts memory rehabilitation outcome after traumatic brain injury. NeuroRehabilitation. (2012) 31:295–310. doi: 10.3233/NRE-2012-0797, 23093456

[16] Sours C Raghavan P Medina AE Roys S Jiang L Zhuo J . Structural and functional integrity of the intraparietal sulcus in moderate and severe traumatic brain injury. J Neurotrauma. (2017) 34:1473–81. doi: 10.1089/neu.2016.4570, 27931179 PMC5385428

[17] Solmaz B Tunç B Parker D Whyte J Hart T Rabinowitz A . Assessing connectivity related injury burden in diffuse traumatic brain injury. Hum Brain Mapp. (2017) 38:2913–22. doi: 10.1002/hbm.23561, 28294464 PMC5426975

[18] Snider SB Bodien YG Frau-Pascual A Bianciardi M Foulkes AS Edlow BL. Ascending arousal network connectivity during recovery from traumatic coma. Neuroimage Clin. (2020) 28:102503. doi: 10.1016/j.nicl.2020.102503, 33395992 PMC7724378

[19] Shah S Yallampalli R Merkley TL McCauley SR Bigler ED Macleod M . Diffusion tensor imaging and volumetric analysis of the ventral striatum in adults with traumatic brain injury. Brain Inj. (2012) 26:201–10. doi: 10.3109/02699052.2012.654591, 22372408

[20] Schmidt AT Lindsey HM Dennis E Wilde EA Biekman BD Chu ZD . Diffusion tensor imaging correlates of resilience following adolescent traumatic brain injury. Cogn Behav Neurol. (2021) 34:259–74. doi: 10.1097/WNN.0000000000000283, 34851864 PMC8647770

[21] Schmidt AT Hanten G Li X Wilde EA Ibarra AP Chu ZD . Emotional prosody and diffusion tensor imaging in children after traumatic brain injury. Brain Inj. (2013) 27:1528–35. doi: 10.3109/02699052.2013.828851, 24266795

[22] Rigon A Voss MW Turkstra LS Mutlu B Duff MC. White matter correlates of different aspects of facial affect recognition impairment following traumatic brain injury. Soc Neurosci. (2019) 14:434–48. doi: 10.1080/17470919.2018.1489302, 29902960 PMC6372351

[23] Rigon A Voss MW Turkstra LS Mutlu B Duff MC. Frontal and temporal structural connectivity is associated with social communication impairment following traumatic brain injury. J Int Neuropsychol Soc. (2016) 22:705–16. doi: 10.1017/S1355617716000539, 27405965 PMC5823263

[24] Rajagopalan V Das A Zhang L Hillary F Wylie GR Yue GH. Fractal dimension brain morphometry: a novel approach to quantify white matter in traumatic brain injury. Brain Imaging Behav. (2019) 13:914–24. doi: 10.1007/s11682-018-9892-2, 29909586

[25] Rabinowitz AR Hart T Whyte J Kim J. Neuropsychological recovery trajectories in moderate to severe traumatic brain injury: influence of patient characteristics and diffuse axonal injury. J Int Neuropsychol Soc. (2018) 24:237–46. doi: 10.1017/S1355617717000996, 29032776 PMC5957498

[26] O'Phelan KH Otoshi CK Ernst T Chang L. Common patterns of regional brain injury detectable by diffusion tensor imaging in otherwise Normal-appearing white matter in patients with early moderate to severe traumatic brain injury. J Neurotrauma. (2018) 35:739–49. doi: 10.1089/neu.2016.4944, 29228858 PMC5831746

[27] Mohamed AZ Cumming P Nasrallah FA Department of Defense Alzheimer’s Disease Neuroimaging Initiative. White matter alterations are associated with cognitive dysfunction decades after moderate-to-severe traumatic brain injury and/or posttraumatic stress disorder. Biol Psychiatry Cogn Neurosci Neuroimaging. (2021) 6:1100–9. doi: 10.1016/j.bpsc.2021.04.014, 33957321

[28] Mofakham S Liu Y Hensley A Saadon JR Gammel T Cosgrove ME . Injury to thalamocortical projections following traumatic brain injury results in attractor dynamics for cortical networks. Prog Neurobiol. (2022) 210:102215. doi: 10.1016/j.pneurobio.2022.102215, 34995694

[29] Lippa SM Yeh PH Ollinger J Brickell TA French LM Lange RT. White matter integrity relates to cognition in service members and veterans after complicated mild, moderate, and severe traumatic brain injury, but not uncomplicated mild traumatic brain injury. J Neurotrauma. (2023) 40:260–73. doi: 10.1089/neu.2022.0276, 36070443

[30] Lippa SM Yeh PH Gill J French LM Brickell TA Lange RT. Plasma tau and amyloid are not reliably related to injury characteristics, neuropsychological performance, or white matter integrity in service members with a history of traumatic brain injury. J Neurotrauma. (2019) 36:2190–9. doi: 10.1089/neu.2018.6269, 30834814 PMC6909749

[31] Kourtidou P McCauley SR Bigler ED Traipe E Wu TC Chu ZD . Centrum semiovale and corpus callosum integrity in relation to information processing speed in patients with severe traumatic brain injury. J Head Trauma Rehabil. (2013) 28:433–41. doi: 10.1097/HTR.0b013e3182585d06, 22832369

[32] Kim J Parker D Whyte J Hart T Pluta J Ingalhalikar M . Disrupted structural connectome is associated with both psychometric and real-world neuropsychological impairment in diffuse traumatic brain injury. J Int Neuropsychol Soc. (2014) 20:887–96. doi: 10.1017/S1355617714000812, 25287217 PMC4275544

[33] Farbota KD Bendlin BB Alexander AL Rowley HA Dempsey RJ Johnson SC. Longitudinal diffusion tensor imaging and neuropsychological correlates in traumatic brain injury patients. Front Hum Neurosci. (2012) 6:160. doi: 10.3389/fnhum.2012.00160, 22723773 PMC3378081

[34] Ewing-Cobbs L DeMaster D Watson CG Prasad MR Cox CS Kramer LA . Post-traumatic stress symptoms after pediatric injury: relation to pre-frontal limbic circuitry. J Neurotrauma. (2019) 36:1738–51. doi: 10.1089/neu.2018.6071, 30672379 PMC6551988

[35] Eisenberg HM Shenton ME Pasternak O Simard JM Okonkwo DO Aldrich C . Magnetic resonance imaging pilot study of intravenous glyburide in traumatic brain injury. J Neurotrauma. (2020) 37:185–93. doi: 10.1089/neu.2019.6538, 31354055 PMC6921286

[36] Dennis EL Rashid F Ellis MU Babikian T Vlasova RM Villalon-Reina JE . Diverging white matter trajectories in children after traumatic brain injury: the RAPBI study. Neurology. (2017) 88:1392–9. doi: 10.1212/WNL.0000000000003808, 28298549 PMC5386434

[37] Dennis EL Jin Y Villalon-Reina JE Zhan L Kernan CL Babikian T . White matter disruption in moderate/severe pediatric traumatic brain injury: advanced tract-based analyses. Neuroimage Clin. (2015) 7:493–505. doi: 10.1016/j.nicl.2015.02.002, 25737958 PMC4338205

[38] Dennis EL Babikian T Alger J Rashid F Villalon-Reina JE Jin Y . Magnetic resonance spectroscopy of fiber tracts in children with traumatic brain injury: a combined MRS - diffusion MRI study. Hum Brain Mapp. (2018) 39:3759–68. doi: 10.1002/hbm.24209, 29749094 PMC6230316

[39] Cox CS Hetz RA Liao GP Aertker BM Ewing-Cobbs L Juranek J . Treatment of severe adult traumatic brain injury using bone marrow mononuclear cells. Stem Cells. (2017) 35:1065–79. doi: 10.1002/stem.2538, 27800660 PMC5367945

[40] Cosgrove ME Saadon JR Mikell CB Stefancin PL Alkadaa L Wang Z . Thalamo-prefrontal connectivity correlates with early command-following after severe traumatic brain injury. Front Neurol. (2022) 13:826266. doi: 10.3389/fneur.2022.826266, 35250829 PMC8895046

[41] Chiou KS Jiang T Chiaravalloti N Hoptman MJ DeLuca J Genova H. Longitudinal examination of the relationship between changes in white matter organization and cognitive outcome in chronic TBI. Brain Inj. (2019) 33:846–53. doi: 10.1080/02699052.2019.1606449 31017479

[42] Chiou KS Genova HM Chiaravalloti ND. Structural white matter differences underlying heterogeneous learning abilities after TBI. Brain Imaging Behav. (2016) 10:1274–9. doi: 10.1007/s11682-015-9497-y, 26699142

[43] Betz J Zhuo J Roy A Shanmuganathan K Gullapalli RP. Prognostic value of diffusion tensor imaging parameters in severe traumatic brain injury. J Neurotrauma. (2012) 29:1292–305. doi: 10.1089/neu.2011.2215 22364596

[44] Bartnik-Olson BL Holshouser B Ghosh N Oyoyo U Nichols J Pivonka-Jones J . Evolving white matter injury following pediatric traumatic brain injury. J Neurotrauma. (2020) 38:111–21. doi: 10.1089/neu.2019.6574, 32515269 PMC7757530

[45] Alivar A Glassen M Hoxha A Allexandre D Yue G Saleh S. Relationship between DTI brain connectivity and functional performance in individuals with traumatic brain injury. Annu Int Conf IEEE Eng Med Biol Soc. (2020) 2020:3256–9. doi: 10.1109/EMBC44109.2020.9176130, 33018699

[46] Shakir A Aksoy D Mlynash M Harris OA Albers GW Hirsch KG. Prognostic value of quantitative diffusion-weighted MRI in patients with traumatic brain injury. J Neuroimaging. (2016) 26:103–8. doi: 10.1111/jon.12286, 26296810

[47] Juranek J Johnson CP Prasad MR Kramer LA Saunders A Filipek PA . Mean diffusivity in the amygdala correlates with anxiety in pediatric TBI. Brain Imaging Behav. (2012) 6:36–48. doi: 10.1007/s11682-011-9140-5, 21979818 PMC3707402

[48] Haber M Amyot F Lynch CE Sandsmark DK Kenney K Werner JK . Imaging biomarkers of vascular and axonal injury are spatially distinct in chronic traumatic brain injury. J Cereb Blood Flow Metab. (2021) 41:1924–38. doi: 10.1177/0271678X20985156, 33444092 PMC8327117

[49] Haber M Amyot F Kenney K Meredith-Duliba T Moore C Silverman E . Vascular abnormalities within Normal appearing tissue in chronic traumatic brain injury. J Neurotrauma. (2018) 35:2250–8. doi: 10.1089/neu.2018.5684, 29609518 PMC6157375

[50] Guerrero-Gonzalez JM Yeske B Kirk GR Bell MJ Ferrazzano PA Alexander AL. Mahalanobis distance tractometry (MaD-tract) - a framework for personalized white matter anomaly detection applied to TBI. NeuroImage. (2022) 260:119475. doi: 10.1016/j.neuroimage.2022.119475, 35840117 PMC9531540

[51] Genova HM Rajagopalan V Chiaravalloti N Binder A Deluca J Lengenfelder J. Facial affect recognition linked to damage in specific white matter tracts in traumatic brain injury. Soc Neurosci. (2015) 10:27–34. doi: 10.1080/17470919.2014.959618, 25223759

[52] Figueiro Longo MG Tan CO Chan ST Welt J Avesta A Ratai E . Effect of transcranial low-level light therapy vs sham therapy among patients with moderate traumatic brain injury: a randomized clinical trial. JAMA Netw Open. (2020) 3:e2017337. doi: 10.1001/jamanetworkopen.2020.17337, 32926117 PMC7490644

[53] Dennis EL Ellis MU Marion SD Jin Y Moran L Olsen A . Callosal function in pediatric traumatic brain injury linked to disrupted white matter integrity. J Neurosci. (2015) 35:10202–11. doi: 10.1523/JNEUROSCI.1595-15.2015, 26180196 PMC4502260

[54] Dennis EL Caeyenberghs K Hoskinson KR Merkley TL Suskauer SJ Asarnow RF . White matter disruption in pediatric traumatic brain injury: results from ENIGMA pediatric moderate to severe traumatic brain injury. Neurology. (2021) 97:e298–309. doi: 10.1212/WNL.0000000000012222, 34050006 PMC8302152

[55] Choi JY Hart T Whyte J Rabinowitz AR Oh SH Lee J . Myelin water imaging of moderate to severe diffuse traumatic brain injury. Neuroimage Clin. (2019) 22:101785. doi: 10.1016/j.nicl.2019.101785, 30927603 PMC6444291

[56] Avesta A Yendiki A Perlbarg V Velly L Khalilzadeh O Puybasset L . Synergistic role of quantitative diffusion magnetic resonance imaging and structural magnetic resonance imaging in predicting outcomes after traumatic brain injury. J Comput Assist Tomogr. (2022) 46:236–43. doi: 10.1097/RCT.0000000000001284, 35297580 PMC8974470

[57] McCrea MA Giacino JT Barber J Temkin NR Nelson LD Levin HS . Functional outcomes over the first year after moderate to severe traumatic brain injury in the prospective, longitudinal TRACK-TBI study. JAMA Neurol. (2021) 78:982–92. doi: 10.1001/jamaneurol.2021.2043, 34228047 PMC8261688

[58] Jochems D van Rein E Niemeijer M van Heijl M van Es MA Nijboer T . Incidence, causes and consequences of moderate and severe traumatic brain injury as determined by abbreviated injury score in the Netherlands. Sci Rep. (2021) 11:19985. doi: 10.1038/s41598-021-99484-6, 34620973 PMC8497630

[59] Castaño-Leon AM Cicuendez M Navarro B Paredes I Munarriz PM Cepeda S . Longitudinal analysis of Corpus callosum diffusion tensor imaging metrics and its association with neurological outcome. J Neurotrauma. (2019) 36:2785–802. doi: 10.1089/neu.2018.5978, 30963801

[60] Castaño-Leon AM Cicuendez M Navarro-Main B Munarriz PM Paredes I Cepeda S . SIXTO OBRADOR SENEC PRIZE 2019: utility of diffusion tensor imaging as a prognostic tool in moderate to severe traumatic brain injury. Part II: longitudinal analysis of DTI metrics and its association with patient's outcome. Neurocirugia. (2020) 31:231–48. doi: 10.1016/j.neucir.2019.11.004, 32035982

[61] Castaño Leon AM Cicuendez M Navarro B Munarriz PM Cepeda S Paredes I . What can be learned from diffusion tensor imaging from a large traumatic brain injury cohort?: white matter integrity and its relationship with outcome. J Neurotrauma. (2018) 35:2365–76. doi: 10.1089/neu.2018.5691, 29786464

[62] Castaño-Leon AM Cicuendez M Navarro-Main B Munarriz PM Paredes I Cepeda S . Sixto Obrador SENEC prize 2019: utility of diffusion tensor imaging as a prognostic tool in moderate to severe traumatic brain injury. Part I. Analysis of DTI metrics performed during the early subacute stage. Neurocirugia. (2020) 31:132–45. doi: 10.1016/j.neucie.2020.02.003 31948842

[63] Grassi DC Zaninotto AL Feltrin FS Macruz FBC Otaduy MCG Leite CDC . Longitudinal whole-brain analysis of multi-subject diffusion data in diffuse axonal injury. Arq Neuropsiquiatr. (2022) 80:280–8. doi: 10.1590/0004-282X-ANP-2020-0595, 35319666 PMC9648927

[64] Grassi DC Zaninotto AL Feltrin FS Macruz FBC Otaduy MCG Leite CC . Dynamic changes in white matter following traumatic brain injury and how diffuse axonal injury relates to cognitive domain. Brain Inj. (2021) 35:275–84. doi: 10.1080/02699052.2020.1859615, 33507820

[65] Jolly AE Bălăeţ M Azor A Friedland D Sandrone S Graham NSN . Detecting axonal injury in individual patients after traumatic brain injury. Brain. (2021) 144:92–113. doi: 10.1093/brain/awaa372, 33257929 PMC7880666

[66] Jolly AE Scott GT Sharp DJ Hampshire AH. Distinct patterns of structural damage underlie working memory and reasoning deficits after traumatic brain injury. Brain. (2020) 143:1158–76. doi: 10.1093/brain/awaa067, 32243506 PMC7174032

[67] McDonald S Dalton KI Rushby JA Landin-Romero R. Loss of white matter connections after severe traumatic brain injury (TBI) and its relationship to social cognition. Brain Imaging Behav. (2019) 13:819–29. doi: 10.1007/s11682-018-9906-0, 29948905

[68] McDonald S Rushby JA Dalton KI Allen SK Parks N. The role of abnormalities in the corpus callosum in social cognition deficits after traumatic brain injury. Soc Neurosci. (2018) 13:471–9. doi: 10.1080/17470919.2017.1356370, 28712330

[69] Verhelst H Giraldo D Vander Linden C Vingerhoets G Jeurissen B Caeyenberghs K. Cognitive training in young patients with traumatic brain injury: a fixel-based analysis. Neurorehabil Neural Repair. (2019) 33:813–24. doi: 10.1177/1545968319868720, 31416407

[70] Verhelst H Vander Linden C De Pauw T Vingerhoets G Caeyenberghs K. Impaired rich club and increased local connectivity in children with traumatic brain injury: local support for the rich? Hum Brain Mapp. (2018) 39:2800–11. doi: 10.1002/hbm.24041, 29528158 PMC6866640

[71] Zaninotto AL Grassi DC Duarte D Rodrigues PA Cardoso E Feltrin FS . DTI-derived parameters differ between moderate and severe traumatic brain injury and its association with psychiatric scores. Neurol Sci. (2022) 43:1343–50. doi: 10.1007/s10072-021-05455-0, 34264413

[72] Yiannakkaras C Konstantinou N Constantinidou F Pettemeridou E Eracleous E Papacostas SS . Whole brain and corpus callosum diffusion tensor metrics: how do they correlate with visual and verbal memory performance in chronic traumatic brain injury. J Integr Neurosci. (2019) 18:95–105. doi: 10.31083/j.jin.2019.02.144, 31321950

[73] Yamagata B Ueda R Tasato K Aoki Y Hotta S Hirano J . Widespread white matter aberrations are associated with phonemic verbal fluency impairment in chronic traumatic brain injury. J Neurotrauma. (2020) 37:975–81. doi: 10.1089/neu.2019.6751, 31631743

[74] Wallace EJ Mathias JL Ward L Pannek K Fripp J Rose S. Chronic white matter changes detected using diffusion tensor imaging following adult traumatic brain injury and their relationship to cognition. Neuropsychology. (2020) 34:881–93. doi: 10.1037/neu0000690, 33197200

[75] Veenith TV Carter EL Grossac J Newcombe VFJ Outtrim JG Nallapareddy S . Normobaric hyperoxia does not improve derangements in diffusion tensor imaging found distant from visible contusions following acute traumatic brain injury. Sci Rep. (2017) 7:12419. doi: 10.1038/s41598-017-12590-2, 28963497 PMC5622132

[76] Van der Eerden AW Van den Heuvel TL Perlbarg V Vart P Vos PE Puybasset L . Traumatic cerebral microbleeds in the subacute phase are practical and early predictors of abnormality of the Normal-appearing white matter in the chronic phase. AJNR Am J Neuroradiol. (2021) 42:861–7. doi: 10.3174/ajnr.A7028, 33632731 PMC8115354

[77] Spitz G Maller JJ O'Sullivan R Ponsford JL. White matter integrity following traumatic brain injury: the association with severity of injury and cognitive functioning. Brain Topogr. (2013) 26:648–60. doi: 10.1007/s10548-013-0283-0, 23532465

[78] Singh K Trivedi R D’souza MM Chaudhary A Khushu S Kumar P . Demonstration of differentially degenerated corpus callosam in patients with moderate traumatic brain injury: with a premise of cortical-callosal relationship. Arch Neurosci. (2015) 2:768. doi: 10.5812/archneurosci.27768

[79] Sihvonen AJ Siponkoski ST Martínez-Molina N Laitinen S Holma M Ahlfors M . Neurological music therapy rebuilds structural connectome after traumatic brain injury: secondary analysis from a randomized controlled trial. J Clin Med. (2022) 11:184. doi: 10.3390/jcm11082184, 35456277 PMC9032739

[80] Scott G Zetterberg H Jolly A Cole JH De Simoni S Jenkins PO . Minocycline reduces chronic microglial activation after brain trauma but increases neurodegeneration. Brain. (2018) 141:459–71. doi: 10.1093/brain/awx339, 29272357 PMC5837493

[81] Scott G Ramlackhansingh AF Edison P Hellyer P Cole J Veronese M . Amyloid pathology and axonal injury after brain trauma. Neurology. (2016) 86:821–8. doi: 10.1212/wnl.0000000000002413, 26843562 PMC4793784

[82] Scott G Hellyer PJ Ramlackhansingh AF Brooks DJ Matthews PM Sharp DJ. Thalamic inflammation after brain trauma is associated with thalamo-cortical white matter damage. J Neuroinflammation. (2015) 12:224. doi: 10.1186/s12974-015-0445-y, 26627199 PMC4666189

[83] Sanchez E El-Khatib H Arbour C Bedetti C Blais H Marcotte K . Brain white matter damage and its association with neuronal synchrony during sleep. Brain. (2019) 142:674–87. doi: 10.1093/brain/awy348, 30698667 PMC6391600

[84] Raizman R Tavor I Biegon A Harnof S Hoffmann C Tsarfaty G . Traumatic brain injury severity in a network perspective: a diffusion MRI based connectome study. Sci Rep. (2020) 10:9121. doi: 10.1038/s41598-020-65948-4, 32499553 PMC7272462

[85] Poudel GR Dominguez DJF Verhelst H Vander Linden C Deblaere K Jones DK . Network diffusion modeling predicts neurodegeneration in traumatic brain injury. Ann Clin Transl Neurol. (2020) 7:270–9. doi: 10.1002/acn3.50984, 32105414 PMC7086000

[86] Pal D Gupta RK Agarwal S Yadav A Ojha BK Awasthi A . Diffusion tensor tractography indices in patients with frontal lobe injury and its correlation with neuropsychological tests. Clin Neurol Neurosurg. (2012) 114:564–71. doi: 10.1016/j.clineuro.2011.12.002, 22209144

[87] Owens JA Spitz G Ponsford JL Dymowski AR Ferris N Willmott C. White matter integrity of the medial forebrain bundle and attention and working memory deficits following traumatic brain injury. Brain Behav. (2017) 7:e00608. doi: 10.1002/brb3.608, 28239519 PMC5318362

[88] Newcombe VF Williams GB Outtrim JG Chatfield D Gulia Abate M Geeraerts T . Microstructural basis of contusion expansion in traumatic brain injury: insights from diffusion tensor imaging. J Cereb Blood Flow Metab. (2013) 33:855–62. doi: 10.1038/jcbfm.2013.11, 23423189 PMC3677102

[89] Newcombe VF Correia MM Ledig C Abate MG Outtrim JG Chatfield D . Dynamic changes in white matter abnormalities correlate with late improvement and deterioration following TBI: a diffusion tensor imaging study. Neurorehabil Neural Repair. (2016) 30:49–62. doi: 10.1177/1545968315584004, 25921349

[90] Navarro-Main B Castaño-León AM Hilario A Lagares A Rubio G Periañez JA . Apathetic symptoms and white matter integrity after traumatic brain injury. Brain Inj. (2021) 35:1043–53. doi: 10.1080/02699052.2021.1953145, 34357825

[91] Molteni E Pagani E Strazzer S Arrigoni F Beretta E Boffa G . Fronto-temporal vulnerability to disconnection in paediatric moderate and severe traumatic brain injury. Eur J Neurol. (2019) 26:1183–90. doi: 10.1111/ene.13963, 30964589

[92] Moen KG Vik A Olsen A Skandsen T Håberg AK Evensen KA . Traumatic axonal injury: relationships between lesions in the early phase and diffusion tensor imaging parameters in the chronic phase of traumatic brain injury. J Neurosci Res. (2016) 94:623–35. doi: 10.1002/jnr.23728, 26948154

[93] Moen KG Håberg AK Skandsen T Finnanger TG Vik A. A longitudinal magnetic resonance imaging study of the apparent diffusion coefficient values in corpus callosum during the first year after traumatic brain injury. J Neurotrauma. (2014) 31:56–63. doi: 10.1089/neu.2013.3000, 23837731 PMC3880061

[94] Mallas E-J De Simoni S Scott G Jolly AE Hampshire A Li LM . Abnormal dorsal attention network activation in memory impairment after traumatic brain injury. Brain. (2021) 144:114–27. doi: 10.1093/brain/awaa380, 33367761

[95] Liang X Yeh CH Domínguez DJF Poudel G Swinnen SP Caeyenberghs K. Longitudinal fixel-based analysis reveals restoration of white matter alterations following balance training in young brain-injured patients. Neuroimage Clin. (2021) 30:102621. doi: 10.1016/j.nicl.2021.102621, 33780865 PMC8022866

[96] Li LM Violante IR Zimmerman K Leech R Hampshire A Patel M . Traumatic axonal injury influences the cognitive effect of non-invasive brain stimulation. Brain. (2019) 142:3280–93. doi: 10.1093/brain/awz252, 31504237 PMC6794939

[97] Leunissen I Coxon JP Caeyenberghs K Michiels K Sunaert S Swinnen SP. Task switching in traumatic brain injury relates to cortico-subcortical integrity. Hum Brain Mapp. (2014) 35:2459–69. doi: 10.1002/hbm.22341, 23913872 PMC6869801

[98] Leunissen I Coxon JP Caeyenberghs K Michiels K Sunaert S Swinnen SP. Subcortical volume analysis in traumatic brain injury: the importance of the fronto-striato-thalamic circuit in task switching. Cortex. (2014) 51:67–81. doi: 10.1016/j.cortex.2013.10.009, 24290948

[99] Lange RT Shewchuk JR Rauscher A Jarrett M Heran MK Brubacher JR . A prospective study of the influence of acute alcohol intoxication versus chronic alcohol consumption on outcome following traumatic brain injury. Arch Clin Neuropsychol. (2014) 29:478–95. doi: 10.1093/arclin/acu027, 24964748

[100] Kurtin DL Violante IR Zimmerman K Leech R Hampshire A Patel MC . Investigating the interaction between white matter and brain state on tDCS-induced changes in brain network activity. Brain Stimul. (2021) 14:1261–70. doi: 10.1016/j.brs.2021.08.004, 34438046 PMC8460997

[101] Kurki TJ Laalo JP Oksaranta OM. Diffusion tensor tractography of the uncinate fasciculus: pitfalls in quantitative analysis due to traumatic volume changes. J Magn Reson Imaging. (2013) 38:46–53. doi: 10.1002/jmri.23901, 23733545

[102] Jolly AE Raymont V Cole JH Whittington A Scott G De Simoni S . Dopamine D2/D3 receptor abnormalities after traumatic brain injury and their relationship to post-traumatic depression. Neuroimage Clin. (2019) 24:101950. doi: 10.1016/j.nicl.2019.101950, 31352218 PMC6664227

[103] Hinson HE Puybasset L Weiss N Perlbarg V Benali H Galanaud D . Neuroanatomical basis of paroxysmal sympathetic hyperactivity: a diffusion tensor imaging analysis. Brain Inj. (2015) 29:455–61. doi: 10.3109/02699052.2014.995229, 25565392 PMC4397147

[104] Håberg AK Olsen A Moen KG Schirmer-Mikalsen K Visser E Finnanger TG . White matter microstructure in chronic moderate-to-severe traumatic brain injury: impact of acute-phase injury-related variables and associations with outcome measures. J Neurosci Res. (2015) 93:1109–26. doi: 10.1002/jnr.23534, 25641684

[105] Graham NSN Jolly A Zimmerman K Bourke NJ Scott G Cole JH . Diffuse axonal injury predicts neurodegeneration after moderate-severe traumatic brain injury. Brain. (2020) 143:3685–98. doi: 10.1093/brain/awaa316, 33099608

[106] Fagerholm ED Hellyer PJ Scott G Leech R Sharp DJ. Disconnection of network hubs and cognitive impairment after traumatic brain injury. Brain. (2015) 138:1696–709. doi: 10.1093/brain/awv075, 25808370 PMC4614120

[107] Drijkoningen D Caeyenberghs K Leunissen I Vander Linden C Leemans A Sunaert S . Training-induced improvements in postural control are accompanied by alterations in cerebellar white matter in brain injured patients. Neuroimage Clin. (2015) 7:240–51. doi: 10.1016/j.nicl.2014.12.006, 25610786 PMC4300016

[108] Diez I Drijkoningen D Stramaglia S Bonifazi P Marinazzo D Gooijers J . Enhanced prefrontal functional–structural networks to support postural control deficits after traumatic brain injury in a pediatric population. Netw Neurosci. (2017) 1:116–42. doi: 10.1162/netn 29911675 PMC5988395

[109] De Simoni S Jenkins PO Bourke NJ Fleminger JJ Hellyer PJ Jolly AE . Altered caudate connectivity is associated with executive dysfunction after traumatic brain injury. Brain. (2018) 141:148–64. doi: 10.1093/brain/awx309, 29186356 PMC5837394

[110] Peng C Xing Y Tao H Yongbing D Jingrui H. Role of diffusion tensor imaging combined with neuron-specific enolase and S100 calcium-binding protein B detection in predicting the prognosis of moderate and severe traumatic brain injury. Iran Red Crescent Med J. (2021) 6:261. doi: 10.32592/ircmj.2021.23.4.261

[111] Castaño-Leon AM Sánchez Carabias C Hilario A Ramos A Navarro-Main B Paredes I . Serum assessment of traumatic axonal injury: the correlation of GFAP, t-tau, UCH-L1, and NfL levels with diffusion tensor imaging metrics and its prognosis utility. J Neurosurg. (2023) 138:454–64. doi: 10.3171/2022.5.JNS22638, 35901687

[112] Castaño-Leon AM Cicuendez M Navarro-Main B Paredes I Munarriz PM Hilario A . Traumatic axonal injury: is the prognostic information produced by conventional MRI and DTI complementary or supplementary? J Neurosurg. (2022) 136:242–56. doi: 10.3171/2020.11.jns203124 34214979

[113] Caeyenberghs K Leemans A Leunissen I Michiels K Swinnen SP. Topological correlations of structural and functional networks in patients with traumatic brain injury. Front Hum Neurosci. (2013) 7:726. doi: 10.3389/fnhum.2013.00726, 24204337 PMC3817367

[114] Caeyenberghs K Leemans A Leunissen I Gooijers J Michiels K Sunaert S . Altered structural networks and executive deficits in traumatic brain injury patients. Brain Struct Funct. (2014) 219:193–209. doi: 10.1007/s00429-012-0494-2, 23232826

[115] Caeyenberghs K Leemans A De Decker C Heitger M Drijkoningen D Vander Linden C . Brain connectivity and postural control in young traumatic brain injury patients: a diffusion MRI based network analysis. Neuroimage Clin. (2012) 1:106–15. doi: 10.1016/j.nicl.2012.09.011, 24179743 PMC3757722

[116] Bourke NJ Yanez Lopez M Jenkins PO De Simoni S Cole JH Lally P . Traumatic brain injury: a comparison of diffusion and volumetric magnetic resonance imaging measures. Brain Commun. (2021) 3:6. doi: 10.1093/braincomms/fcab006, 33981994 PMC8105496

[117] Boccia M Barbetti S Valentini F De Angelis C Tanzilli A Fabio V . Neural underpinnings of the slowness of information processing in patients with traumatic brain injury: insights from tract-based spatial statistics. Neurol Sci. (2022) 43:5083–6. doi: 10.1007/s10072-022-06150-4, 35583841. 35583841

[118] Bazeed MF El-Fatah Ghanem MA Afif HFS Sunbulli MHA Abdelaal AME. Can diffusion tensor imaging predict motor power affection after moderate traumatic brain injury? Egypt J Radiol Nucl Med. (2013) 44:879–83. doi: 10.1016/j.ejrnm.2013.09.006

[119] Adnan A Crawley A Mikulis D Moscovitch M Colella B Green R. Moderate-severe traumatic brain injury causes delayed loss of white matter integrity: evidence of fornix deterioration in the chronic stage of injury. Brain Inj. (2013) 27:1415–22. doi: 10.3109/02699052.2013.823659, 24102365

[120] Baxter D Sharp DJ Feeney C Papadopoulou D Ham TE Jilka S . Pituitary dysfunction after blast traumatic brain injury: The UK BIOSAP study. Ann Neurol. (2013) 74:527–36. doi: 10.1002/ana.23958 23794460 PMC4223931

[121] Yang A Xiao XH Liu ZH Wan ZL Wang ZY. A multimodal magnetic resonance imaging study of recovery of consciousness in severe traumatic brain injury: preliminary results. J Neurotrauma. (2018) 35:308–13. doi: 10.1089/neu.2017.5335, 29141511

[122] Ubukata S Ueda K Sugihara G Yassin W Aso T Fukuyama H . Corpus callosum pathology as a potential surrogate marker of cognitive impairment in diffuse axonal injury. J Neuropsychiatry Clin Neurosci. (2016) 28:97–103. doi: 10.1176/appi.neuropsych.15070159, 26569151

[123] Simeone P Auzias G Lefevre J Takerkart S Coulon O Lesimple B . Long-term follow-up of neurodegenerative phenomenon in severe traumatic brain injury using MRI. Ann Phys Rehabil Med. (2022) 65:101599. doi: 10.1016/j.rehab.2021.101599, 34718191

[124] Sener S Van Hecke W Feyen BF Van der Steen G Pullens P De Van Hauwe L . Diffusion tensor imaging: a possible biomarker in severe traumatic brain injury and aneurysmal subarachnoid hemorrhage? Neurosurgery. (2016) 79:786–93. doi: 10.1227/NEU.0000000000001325, 27352277

[125] Puybasset L Perlbarg V Unrug J Cassereau D Galanaud D Torkomian G . Prognostic value of global deep white matter DTI metrics for 1-year outcome prediction in ICU traumatic brain injury patients: an MRI-COMA and CENTER-TBI combined study. Intensive Care Med. (2022) 48:201–12. doi: 10.1007/s00134-021-06583-z, 34904191

[126] Palacios EM Sala-Llonch R Junque C Roig T Tormos JM Bargallo N . Resting-state functional magnetic resonance imaging activity and connectivity and cognitive outcome in traumatic brain injury. JAMA Neurol. (2013) 70:845–51. doi: 10.1001/jamaneurol.2013.38, 23689958

[127] Palacios EM Sala-Llonch R Junque C Roig T Tormos JM Bargallo N . White matter integrity related to functional working memory networks in traumatic brain injury. Neurology. (2012) 78:852–60. doi: 10.1212/WNL.0b013e31824c465a, 22345222

[128] Palacios EM Sala-Llonch R Junque C Fernandez-Espejo D Roig T Tormos JM . Long-term declarative memory deficits in diffuse TBI: correlations with cortical thickness, white matter integrity and hippocampal volume. Cortex. (2013) 49:646–57. doi: 10.1016/j.cortex.2012.02.011, 22482692

[129] Magnoni S Mac Donald CL Esparza TJ Conte V Sorrell J Macrì M . Quantitative assessments of traumatic axonal injury in human brain: concordance of microdialysis and advanced MRI. Brain. (2015) 138:2263–77. doi: 10.1093/brain/awv152, 26084657 PMC4840950

[130] Lesimple B Caron E Lefort M Debarle C Pélégrini-Issac M Cassereau D . Long-term cognitive disability after traumatic brain injury: contribution of the DEX relative questionnaires. Neuropsychol Rehabil. (2020) 30:1905–24. doi: 10.1080/09602011.2019.1618345, 31116085

[131] Laouchedi M Galanaud D Delmaire C Fernandez-Vidal S Messé A Mesmoudi S . Deafferentation in thalamic and pontine areas in severe traumatic brain injury. J Neuroradiol. (2015) 42:202–11. doi: 10.1016/j.neurad.2014.03.001, 24997478

[132] Galanaud D Perlbarg V Gupta R Stevens RD Sanchez P Tollard E . Assessment of white matter injury and outcome in severe brain trauma: a prospective multicenter cohort. Anesthesiology. (2012) 117:1300–10. doi: 10.1097/ALN.0b013e3182755558, 23135261

[133] Ferraro S Nigri A Nava S Rosazza C Sattin D Sebastiano DR . Interhemispherical anatomical disconnection in disorders of consciousness patients. J Neurotrauma. (2019) 36:1535–43. doi: 10.1089/neu.2018.5820, 30520674

[134] Dinkel J Drier A Khalilzadeh O Perlbarg V Czernecki V Gupta R . Long-term white matter changes after severe traumatic brain injury: a 5-year prospective cohort. AJNR Am J Neuroradiol. (2014) 35:23–9. doi: 10.3174/ajnr.A3616, 23846796 PMC7966468

[135] Debarle C Perlbarg V Jacquens A Pélégrini-Issac M Bisch M Prigent A . Global mean diffusivity: a radiomarker discriminating good outcome long term after traumatic brain injury. Ann Phys Rehabil Med. (2021) 64:101433. doi: 10.1016/j.rehab.2020.08.002, 32992024

[136] Enciso-Olivera CO Ordóñez-Rubiano EG Casanova-Libreros R Rivera D Zarate-Ardila CJ Rudas J . Structural and functional connectivity of the ascending arousal network for prediction of outcome in patients with acute disorders of consciousness. Sci Rep. (2021) 11:22952. doi: 10.1038/s41598-021-98506-7, 34824383 PMC8617304

[137] Andreasen SH Andersen KW Conde V Dyrby TB Puonti O Kammersgaard LP . Limited Colocalization of microbleeds and microstructural changes after severe traumatic brain injury. J Neurotrauma. (2020) 37:581–92. doi: 10.1089/neu.2019.6608, 31588844

[138] Abe H Shimoji K Nagamine Y Fujiwara S Izumi SI. Predictors of recovery from traumatic brain injury-induced prolonged consciousness disorder. Neural Plast. (2017) 2017:9358092. doi: 10.1155/2017/9358092, 28326199 PMC5343264

[139] Vachha B Huang SY. MRI with ultrahigh field strength and high-performance gradients: challenges and opportunities for clinical neuroimaging at 7 T and beyond. Eur Radiol Exp. (2021) 5:35. doi: 10.1186/s41747-021-00216-2, 34435246 PMC8387544

[140] Soares JM Marques P Alves V Sousa N. A hitchhiker's guide to diffusion tensor imaging. Front Neurosci. (2013) 7:31. doi: 10.3389/fnins.2013.00031, 23486659 PMC3594764

[141] Jones DK. The effect of gradient sampling schemes on measures derived from diffusion tensor MRI: a Monte Carlo study. Magn Reson Med. (2004) 51:807–15. doi: 10.1002/mrm.20033, 15065255

[142] Fortin J-P Parker D Tunç B Watanabe T Elliott MA Ruparel K . Harmonization of multi-site diffusion tensor imaging data. NeuroImage. (2017) 161:149–70. doi: 10.1016/j.neuroimage.2017.08.047, 28826946 PMC5736019

[143] Kochunov P Jahanshad N Sprooten E Nichols TE Mandl RC Almasy L . Multi-site study of additive genetic effects on fractional anisotropy of cerebral white matter: comparing meta and megaanalytical approaches for data pooling. NeuroImage. (2014) 95:136–50. doi: 10.1016/j.neuroimage.2014.03.033, 24657781 PMC4043878

[144] Mueller BA Lim KO Hemmy L Camchong J. Diffusion MRI and its role in neuropsychology. Neuropsychol Rev. (2015) 25:250–71. doi: 10.1007/s11065-015-9291-z, 26255305 PMC4807614

[145] Borja MJ Chung S Lui YW. Diffusion MR imaging in mild traumatic brain injury. Neuroimaging Clin N Am. (2018) 28:117–26. doi: 10.1016/j.nic.2017.09.009, 29157848 PMC9034763

