# Supplementary material for: The second decade of DTI in TBI Part 2: a systematic review of moderate and severe TBI
Source: Front Neurol. 2026 Feb 6;17:1734550. doi: 10.3389/fneur.2026.1734550 (PMC12920199; doi:10.3389/fneur.2026.1734550)
Supplement: Supplementary file 1 [file Data_Sheet_1.PDF]

**Supplemental Materials**. Full Literature Search Strategies

| Database | PubMed |
|---|---|
| Date | 09/28/2022 |

| Line # | Search Terms |
|---|---|
| 1 | "Diffusion Tensor Imaging"[Mesh] OR "Diffusion Magnetic Resonance Imaging"[Mesh] OR “diffusion tensor imaging”[tiab] OR “DTI”[tiab] |
| 2 | "Brain Injuries, Traumatic"[Mesh] OR “traumatic brain injur*”[tiab] OR “TBI”[tiab] OR “concussion*”[tiab] |
| 3 | #1 AND #2 |
| 4 | #3 NOT ("Muridae"[Mesh] OR “mouse”[tiab] OR “mice”[tiab] OR “rat”[tiab] OR "rats"[tiab] OR “rodent*”[tiab]) |
| 5 | #4 NOT ("meta-analysis"[PT] OR "systematic review"[PT] OR "review"[PT] OR "case reports"[PT] OR "editorial"[PT]) |
| **Filters** | English-language only, publication date 2012 to 2022 |
| **# of Results** | **671** |

| Database | Embase |
|---|---|
| Date | 09/28/2022 |

| Line # | Search Terms |
|---|---|
| 1 | 'diffusion tensor imaging'/exp OR ‘diffusion tensor imaging’:ti,ab OR ‘DTI’:ti,ab |
| 2 | 'traumatic brain injury'/exp OR ‘traumatic brain injur*’:ti,ab OR ‘TBI’:ti,ab OR ‘concussion*’:ti,ab |
| 3 | #1 AND #2 |
| 4 | #3 NOT ('Muridae'/exp OR ‘mouse’:ti,ab OR ‘mice’:ti,ab OR ‘rat’:ti,ab OR ‘rats’:ti,ab OR ‘rodent*’:ti,ab) |
| 5 | #4 NOT ('conference abstract'/it OR ‘conference paper’/it OR ‘review’/it OR ‘editorial’/it OR 'case report'/de OR ‘case report’/de OR ‘systematic review’/de OR ‘meta analysis’/de) |
| **Filters** | English-language only, publication date 2012 to 2022 |
| **# of Results** | **714** |

| Database | Cochrane Library |
|---|---|
| Date | 09/28/2022 |

| Line # | Search Terms |
|---|---|
| 1 | “diffusion tensor imaging” OR “DTI” OR “diffusion magnetic resonance imaging” OR “diffusion MRI” |
| 2 | “traumatic brain injur*” OR “TBI” OR “concussion*” |
| 3 | #1 AND #2 |
| 4 | #3 NOT (“mouse” OR “mice” OR “rat” OR "rats" OR “rodent*”) |
| **Filters** | Publication date 2012 to 2022 |
| **# of Results** | **35** |

| Database | Web of Science |
|---|---|
| Date | 09/28/2022 |

| Line # | Search Terms |
|---|---|
| 1 | TI=("diffusion tensor imaging" OR "DTI" OR "diffusion magnetic resonance imaging" OR "diffusion MRI") |
| 2 | AB=("diffusion tensor imaging" OR "DTI" OR "diffusion magnetic resonance imaging" OR "diffusion MRI") |
| 3 | #1 OR #2 |
| 4 | TI=("traumatic brain injur*" OR "TBI" OR "concussion*") |
| 5 | AB=("traumatic brain injur*" OR "TBI" OR "concussion*") |
| 6 | #4 OR #5 |
| 7 | #3 AND #6 |
| 8 | ALL=("mouse" OR "mice" OR "rat" OR "rats" OR "rodent*") |
| 9 | #7 NOT #8 |
| **Filters** | English-language only, publication date 2012 to 2022, document type = article |
| **# of Results** | **487** |
